# Ionomer-Free NiFe/NiFeO Bilayer Oxygen Evolution Reaction Electrocatalyst Prepared by a Magnetron Sputtering at Oblique Angle Bottom-Up Deposition Method

**DOI:** 10.1021/acscatal.5c04915

**Published:** 2025-10-17

**Authors:** José Manuel Luque-Centeno, Álvaro Carmo-Delcán, Mikel Martínez-Olaizola, Celia Gómez-Sacedón, Antonio de Lucas-Consuegra, Agustín R. González-Elipe, Francisco Yubero, José Javier Brey Sánchez, Jorge Gil-Rostra

**Affiliations:** † Laboratory of Nanotechnology on Surfaces and Plasma, Institute of Materials Science of Seville (CSIC-Universidad de Sevilla), Av. Américo Vespucio 49, E-41092 Sevilla, Spain; ‡ Department of Chemical Engineering, School of Chemical Sciences and Technologies, 16733 University of Castilla-La Mancha, Avda. Camilo José Cela 12, E-13071 Ciudad Real, Spain; § H2B2 Electrolysis Technologies, Moriscas 46-48, Pol. Ind. La Isla. Dos Hermanas, E-41703 Sevilla, Spain; ∥ Department of Engineering, Universidad Loyola Andalucía, Avenida de Las Universidades s/n, Dos Hermanas, E-41704 Sevilla, Spain

**Keywords:** bilayer electrocatalysts, magnetron sputtering, NiFe catalysts, anion exchange membrane water electrolysis, oxygen evolution reaction, layered double hydroxide, hydrogen

## Abstract

This manuscript reports on a Ni/Fe-based bilayer catalyst developed to boost the oxygen evolution reaction in anion exchange membrane water electrolyzers. The electrochemical behavior toward the oxygen evolution reaction of several NiFe/NiFeO metal–oxide bilayer catalysts, prepared by magnetron sputtering at oblique angle deposition (MS-OAD) on a flat stainless-steel substrate, was assessed in a three-electrode electrochemical cell in comparison with the behavior of both a metal NiFe and an oxide NiFeOx single-layer catalyst. The morphology and chemical nature of these catalysts, as prepared and after electrochemical usage, were characterized by X-ray photoelectron spectroscopy, Raman spectroscopy, Fourier transform infrared spectroscopy, and scanning electron microscopy. A thorough electrochemical characterization of the different catalyst formulations revealed a higher efficiency for the bilayer catalysts, in terms of both activity and long-term stability, and provided some clues to account for this superior performance in terms of morphology and surface reactivity of each catalyst. As a proof of concept, the best-performing bilayer configuration was then deposited onto a stainless steel felt porous transport layer (PTL) substrate and tested as an ionomer-free anode electrode in a membrane electrode assembly (MEA). Results revealed that the MS-OAD catalysts performed well when deposited on PTLs and that, under this configuration, a bilayer catalyst anode is slightly more efficient than the NiFe single-layer catalyst. Additionally, the possibility of scaling up the MS-OAD procedure to large areas has been demonstrated by the preparation of the bilayer catalysts on a 64 cm^2^ PTL and its successful integration and operation in a large prototype single cell.

## Graphical abstract

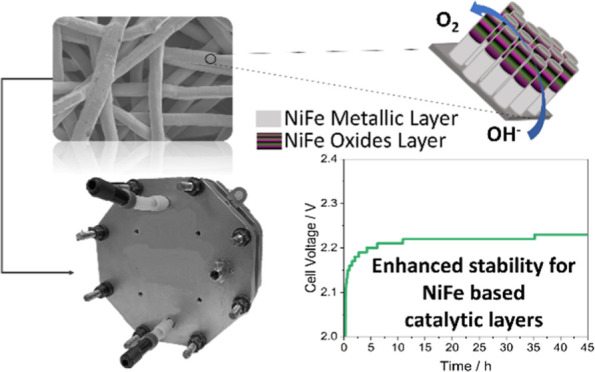

## 1 Introduction

Green hydrogen, produced by renewable energy-powered electrochemical water splitting, has been postulated as a reliable energy carrier due to its cleanliness and high energy density compared to traditional energy sources based on fossil fuels.^1,2^ Nowadays, the most widespread technology for obtaining green hydrogen is water electrolysis using proton exchange membrane (PEM) electrolyzers, which require noble metal catalysts such as Pt for the hydrogen evolution reaction (HER) and Ir or Ru oxides for the oxygen evolution reaction (OER).^3^ The scarcity and high cost of these elements have fostered the search for alternatives to PEM technologies, among which anion exchange membrane (AEM) water electrolysis is a promising option. AEM water electrolysis (AEMWE) can be driven with non-noble metal-based electrocatalysts both for OER and HER, whose kinetics are enhanced in alkaline media and show great potential for use with seawater.^4^ An additional advantage is that alkaline or neutral media reduce the corrosion of the stack components and enhance the stability of catalysts based on non-noble elements.^5^ The most popular AEMWE catalysts for the OER incorporate 3d transition metals such as Co, Ni, Fe, or Mn, whose abundance, high electrocatalytic activity, and low cost make them a good alternative to “platinum group metals” (PGMs)-based catalysts.^6,7^ In particular, many studies have focused on Ni and Fe-based formulations, including their alloys and compounds such as hydroxides and oxides,^8–10^ sulfides,^11^ and phosphides.^12,13^ In general, NiFe (oxy)­hydroxides (NiFeOOH) are recognized as some of the best catalytic options for OER to date. This attribution has been supported by the fact that transition metal compounds such as nitrides, sulfides, or phosphides are not thermodynamically stable in alkaline media and during operation at high current densities eventually oxidize into oxides or hydroxides.^14,15^ The catalytic activity of NiFe (oxy)­hydroxides depends on physicochemical properties such as their crystalline structure, Ni/Fe ratio, oxygen vacancies, and related features. For example, it is known that a platelet-like morphology of these catalysts contributes to the formation of active sites toward OER.^16^ Evidence in this regard has fostered the development of processing methods for the fabrication of layered double hydroxide (LDH) structures with an outstanding catalytic behavior for OER. Procedures include coprecipitation,^17^ ball milling,^18^ hydrothermal synthesis,^19^ or electrochemical oxidation of NiFe alloys.^20^ However, these synthesis methods entail the use of potentially polluting solvents, require long synthesis times, and involve several steps that could hinder the reproducibility, homogeneity, and scalability of the prepared catalyst.^21^ Moreover, long term stability of catalysts Moreover, long term satability ofstsbased on these structures can be an issue, as suggested by the results of the present work. Addressing these problems, we and other researchers have claimed the use of dry synthesis methods such as magnetron sputtering (MS), a physical vapor deposition technique that allows the one-step bottom-up preparation of catalyst-coated electrodes.^22–26^ The MS deposition method does not require liquid reagents and solvents, and its energy requirements are low and produce no waste, factors ensuring the environmentally friendly character of this preparation method of electrodes. Additionally, the MS technique enables the modification of the electrodes’ nanoarchitecture by adjusting the sputtering conditions during the deposition procedure. Thus, following a bottom-up approach, it is possible to control the catalyst layer thickness and morphology, as well as in-depth tailoring the composition, microstructure, porosity, conductivity, hydrophilicity, or the presence of specific functional groups from the support/catalyst to the catalyst/membrane interface.^27,28^ In addition, using MS in an oblique angle deposition (OAD) geometry leverages the fabrication of highly porous catalysts, while still preserving a high conformality onto irregular catalyst supports such as fiber-based carbon cloths, fibers, or metal felts, commonly used as porous transport layers (PTLs) in industrial AEMWE cells.^29–31^

Despite their good catalytic properties, NiFe (oxy)­hydroxides present some drawbacks, such as a complicated and low reproducible synthesis procedure,^32^ a low electrical conductivity, limitations in the number of active sites, and a poor stability during long-term electrolysis.^33,34^ To cope with some of these issues, certain authors have suggested combining NiFe (oxy)­hydroxides with NiFe oxides to lower the catalyst system resistivity, improve the formation of redox active metal centers to increase the adsorption of intermediate species, and maximize their activity.^35,36^ Within these premises, in this work, we propose the use of magnetron sputtering in oblique angle deposition configuration (MS-OAD) to deposit NiFeOx/NiFe catalyst bilayers on PTL supports (i.e., electrodes with oxide/metal/PTL architectures) to improve the electrochemical OER performance of AEMWE cells.

Two features are to be highlighted in the developed approach: (i) the preparation of an oxidized Ni–Fe phase (NiFeOx) using reactive MS conditions (i.e., a O_2_/Ar plasma discharge) during the deposition and (ii) the fabrication of a bilayer catalyst coating, with a first bottom part consisting of a Ni–Fe metallic layer with high electrical conductivity and a low resistance Ohmic contact with the PTLs and a second NiFeOx layer at the top. The thickness of this second layer has been varied as a running parameter to optimize the electrode performance after in situ electrochemical conditioning to obtain the NiFe-oxyhydroxide (NiFe-OOH) phase. For comparison purposes, single metallic NiFe and single oxide NiFeOx catalyst (9:1 atomic ratio) layers have also been studied. The morphology and chemical nature of the catalysts (in their “as-prepared” and “used” states) have been characterized by X-ray photoelectron spectroscopy (XPS), Raman spectroscopy, X-ray diffraction (XRD), Fourier transform infrared spectroscopy (FTIR), and scanning electron microscopy (SEM). The electrochemical behavior and the OER performance of the catalysts deposited onto a flat stainless-steel plate have been first assessed in a three-electrode half-cell using a variety of electrochemical methods.

As a proof of concept of the possibilities of the MS-OAD technique for the preparation of catalysts, the best bilayer formulation was then deposited onto a porous stainless steel felt acting as a PTL and tested as an anode in a full-cell with a membrane electrode assembly (MEA) configuration working without ionomers.^25,37^ The objective of this exploratory work has been to demonstrate that MS-OAD catalysts are compatible with cell membrane operation and that, under these conditions, a bilayer configuration is superior in performance to single metallic or oxide layer catalysts. Results confirmed that anodes incorporating NiFeOx/NiFe bilayer catalysts, even in the absence of ionomers, presented a slightly higher electrochemical yield and a more stable behavior (less degradation) than those made of a single NiFe layer used as reference. Additionally, preliminary results with a cell of a relevant electrode area of 64 cm^2^ intended for industrial applications, including the assessment of the Faradaic efficiency for the production of hydrogen, prove that MS-OAD catalysts can be efficiently prepared at large scales and sustain the capacity of this technique for the manufacturing of electrodes for commercial purposes.

## 2 Methods

### 2.1 Catalyst Substrates

Stainless-steel (SS316L) plates and felts, as well as pieces of polished silicon wafers (6 in. diameter, 675 μm thick, orientation (100), supplied by Siegert Wafers GmbH), were employed as substrates for the deposition of the catalytic layers. Stainless-steel plates consisted of a 316L quality steel of 2.5 × 2.5 cm size purchased from ACERINOX EUROPE. The felts consisted of 12 μm diameter sintered fibers with a density of 675 g/cm^2^, 70% porosity, and a thickness of 280 μm (ref 20FP3, from BEKAERT). Pieces of stainless-steel plates, felts, and undoped silicon wafers were located at equivalent locations within the sample holder of the catalyst deposition system in each deposition run.

Anode catalyst samples prepared on stainless-steel plates were electrochemically evaluated in a three-electrode cell. The stainless-steel felts were used as PTLs for evaluating the electrocatalyst in a full-cell MEA configuration. Finally, the silicon wafers were used as control substrates to estimate by cross-sectional SEM analysis the so-called equivalent thickness of the deposited catalyst layer. Note that the equivalent thicknesses of the catalyst layers considered in this work are well below the diameter of the PTL felt fibers and that this parameter is an indirect way to estimate and normalize the catalyst load in each prepared sample.

All substrates were cleaned with acetone, isopropyl alcohol, and water using an ultrasonic bath to remove impurities and dust. In addition, stainless-steel plates and felts were treated with a 10% nitric acid + 0.5% ammonium fluoride solution to generate some surface pickling and remove possible impurities and oxidation layers from their surfaces. Figure S1 in the Supporting Information file shows SEM images of the stainless-steel plate and felt substrates used as catalyst supports before and after the cleaning and pickling treatment.

### 2.2 Anode Catalysts

Following the proposed MS-OAD bottom-up fabrication approach, metallic NiFe single-layer, oxide NiFeOx single-layer, and NiFe/NiFeOx bilayer catalysts have been prepared and tested as anodes.

As a common feature, deposition of these catalytic layers was performed from a metallic sputtering target with a NiFe alloy composition (Ni:Fe atomic ratio of 9:1, 99.99% purity, Testbourne Ltd.). The selected Ni:Fe ratio was identified in previous studies as the optimal choice for maximizing the NiFe system efficiency.^23^ The deposition was carried out in a vacuum chamber (base pressure ∼2 × 10^–6^ mbar) at room temperature. The experimental MS-OAD setup has been described in previous works.^37^ In short, a two inch circular magnetron head (high-strength magnetics SW50 from Gencoa Ltd.) operated with a pulsed DC power supply (Pinnacle+ from Advanced Energy) was used as a sputtering source. Before any deposition, a presputtering treatment (50 W, 120 kHz, 5 × 10^–3^ mbar, Ar gas) was applied to clean the target. Several substrates were placed on a circular rotatable sample holder (10 cm diameter) located at ∼90° with respect to the axis of the magnetron target. The sample holder was axially rotated at a rate of ∼5 rpm during the deposition process. This configuration ensures a homogeneous distribution of catalyst loads onto the supports.

Metallic NiFe single-layer catalysts were grown (∼800 nm equivalent thickness) using an Ar discharge (150 W, 120 kHz, 30 sccm) at a pressure of 5 × 10^–3^ mbar. The deposition rate was ∼20 nm·min^–1^. These samples have been labeled as *NiFe*.

Oxide NiFeOx single-layer catalysts were grown (∼1100 nm equivalent thickness) using the same process conditions as before, but with an even Ar/O_2_ ratio (15 sccm each gas) in the plasma discharge. The deposition rate was ∼4 nm·min^–1^. These samples have been labeled as *NiFeOx*.

Metal/oxide bilayer catalysts were prepared with a fixed equivalent thickness for the metallic layer (∼800 nm) and a varying equivalent thickness of the oxide layer. The interlayertransition between the metal and the oxide layers was made through a metal/oxide gradient layer, keeping fixed the power conditions while increasing by 5 sccm the oxygen flow and decreasing the Ar flow in an equivalent way every 5 min to reach a 15:15 sccm Ar/O_2_ flow ratio in the plasma discharge. Three NiFe/NiFeO bilayer catalysts with equivalent oxide layer thicknesses of ∼200, ∼500, and ∼800 nm, respectively, were prepared. These bilayer catalyst samples have been labeled as *NiFe/NiFeOx-200*, *NiFe/NiFeOx-500*, and *NiFe/NiFeOx-800*, respectively.

### 2.3 Physicochemical Characterization

The study of the catalyst layers’ morphology deposited on the different SS substrates and the determination of their equivalent thicknesses were carried out by Scanning Electron Microscopy (SEM) using a Hitachi S4800 field-emission microscope, operated at 2 keV. Top view and cross-section images were taken. The samples deposited onto undoped silicon wafer were also analyzed by transmittance Fourier Transform Infrared (FT-IR) spectroscopy using a JASCO FT/IR-6200 IRT-5000 instrument. The structural analysis of the prepared layers was performed by X-ray Diffraction (XRD) using a Panalytical X’PERT PRO device.

The surface chemical composition of the deposited samples was also determined by X-ray Photoelectron Spectroscopy (XPS) using a SPECS PHOIBOS 100-DLD photoelectron spectrometer operated with Mg Kα radiation as the excitation source and 20 eV pass energy. In addition, Raman spectroscopy was employed to further investigate the chemical composition of the catalytic layer. For this purpose, a LabRAM Horiba Jobin Yvon (532 nm laser) was employed. These analyses were carried out for the as-deposited, conditioned, and aged samples (see below).

### 2.4 Electrochemical Conditioning and Measurements in the Three-Electrodes Cell

The electrocatalytic behavior of the single-layer and bilayer catalyst samples deposited on stainless-steel plates was studied in a three-electrode cell (Redox.me) connected to a potentiostat (Metrohm AutoLab workstation PGSTAT302N). The catalyst samples were used as working electrodes, and a 2 × 1 cm carbon cloth was used as a counter electrode. A Hg/HgO electrode filled with a 1.0 M KOH electrolyte solution was employed as a reference electrode. All the voltage measurements were referred to the potential of the reversible hydrogen electrode (RHE). The geometric area of the working electrode in contact with the electrolyte was 1.0 cm^2^ (Figure S2 in Supporting Information 2).

The as-prepared catalysts were conditioned by a cycling voltammetry (CV) procedure. It consisted of 200 reversible *i*–*V* scans (i.e., 200 cycles) varying the voltage between 0.78 and 1.63 V versus RHE at a scanning rate of 0.02 V/s in a 1.0 M KOH solution bubbled with nitrogen gas. The catalytic activity of the conditioned catalysts was determined by linear sweep voltammetry (LSV) from 0.78 to 1.73 V vs RHE at 0.005 V/s, while their stability was assessed by an aging procedure consisting of a chronopotentiometry (CP) test at a fixed current of 0.025 A·cm^–2^ during 12 h. Electrochemical impedance spectroscopy (EIS) analysis was performed between 1.23 and 1.53 V vs RHE, with a 10 mV perturbation potential within the 100 kHz to 100 mHz frequency range. All of the measurements were done at room temperature. All the potentials measured in the performed tests have been corrected using the electrolyte’s resistance determined by EIS.

The electrochemical active surface area (ECSA) values of the samples were obtained using a CV method.^38^ Measurements were performed between 0.88 and 1.08 V (vs RHE) at 0.1, 0.08, 0.06, 0.04, and 0.02 V/s. By representing the average current at 0.98 V (vs RHE) against the scan rate, a straight line is obtained, whose slope equals the electrochemical double-layer capacitance *C*_dl_. Then ECSA values were determined using the following equation:

1 $$ECSA=\frac{C_{dl}}{C_{S}}$$

where *C*_S_ is the specific capacitance of the SS316L plate used as the catalyst support (in our case, *C*_S_ = 0.022 mF·cm^–2^).

### 2.5 Proofs of Concepts in a Single Cell with a MEA Configuration

Anode catalyst samples with the best electrochemical performance, as determined in the three-electrode cell, were tested as anodes in membrane electrode assemblies. In these studies aiming at verifying the possibilities of MS-OAD for the manufacturing of catalytic anodes for single-cell integration, nonoptimized Ni catalytic layers (540 nm equivalent thickness) deposited by MS-OAD on the same stainless-steel felt were used as cathode electrodes (cf. more details can be found in previous works^23,25^ ). Commercial Fumasep FAA-3-50 membranes (Fumatech) were used as anion exchange membranes of the MEA. Tests were carried out using two different single-cell MEA devices with either 5 or 64 cm^2^ electrode areas. The first was a commercial AEM cell from Dioxide Materials, and the second was a homemade single cell developed in collaboration with H2B2 Electrolysis Technologies. Cells were closed and sealed with screws and bolts using 6 and 25 N m torque for the 5 and 64 cm^2^ electrode area cells, respectively. These conditions ensured proper electrical connectivity among the cell elements while preventing water leaks. Membranes were conditioned in a 1.0 M KOH solution for 24 h, following the provider’s instructions.

Both cells were operated in dry-cathode mode (the electrolyte only flows through the anode compartment, and water reaches the cathode by permeation through the membrane) according to the working conditions employed in commercial electrolyzers. To characterize the electrochemical behavior of the anodic catalysts layers, a 0.1 M KOH solution was circulated in the anode chamber (30 ml·min^–1^ flow rate for 5 cm^2^ and 100 ml·min^–1^ for a 64 cm^2^ cell) using a peristaltic pump (pump drive 5001, Heidolph) with a closed-loop configuration (batch operation mode). The voltage/intensity response of the cells was studied by using an ELECTROAUTOMATIKS PS9080 power supply.

The MEA integration and electrochemical performance of the anode catalysts were determined with the small single cell (5 cm^2^) by registering voltage–intensity curves from 0 to 1.6 A·cm^–2^ applied current. Stability tests were performed by means of chronopotentiometric measurements at current densities of 0.1 and 0.6 A·cm^–2^. The charge transfer resistance of the complete cells was determined by EIS using a perturbation potential of 10 mV amplitude and AC frequencies from 4.0 kHz to 10.0 mHz using a 10 A current booster coupled to the potentiostat (Metrohm AutoLab workstation PGSTAT302N). For these measurements, cutoff values of 2.5 V and 1 A·cm^–2^ have been set up as safety limits.

The compatibility of the MS-OAD technique to prepare catalysts on large-area PTL supports was tested in a 64 cm^2^ electrode area cell. Here, *i*–*V* polarization curves were recorded using the same protocol as that employed before for the 5 cm^2^ cell. Instead, the stability test of this device was performed during 5 days with 5 hours of continuous operation each day to simulate solar-driven production. Additionally, cutoff limits were adjusted to 2.2 V and 1 A·cm^–2^, following the recommendation found in different harmonized protocols for AEMWE testing.^39,40^ Hydrogen production was measured using two mass flow sensors from Bronkhorst company, calibrated for H_2_ with two measuring ranges, from 0 to 65 ml/min and 0 to 500 ml/min, referred to normal conditions of pressure and temperature (1 atm, 293.15 K). The Faradaic efficiency (*E*_F_) for hydrogen production was calculated by comparing the actual volume (*V*_R_) of hydrogen produced to the theoretical one. Faradaic hydrogen volume (*V*_T_) has been calculated using the following equation:

2 $$V_{T}=\frac{I\cdot t}{2e^{-}\cdot F}\cdot 22.4\cdot 1000\;\left(ml\right)$$

where *I* is the applied current, *t* is the time of measure (1 min), and *F* is the Faraday constant. The obtained value is employed to determine the efficiency of *E*_F_ by the formula:

3 $$E_{F}=\frac{V_{R}}{V_{T}}\cdot 100$$

## 3 Results and Discussion

### 3.1 Physicochemical Characterization and Electrochemical Conditioning of Catalytic Layers on SS Plates

The SEM cross-section images of the as-prepared catalyst coatings deposited on polished silicon wafer pieces, included in Figure S3 of Supporting Information 3, depicted a well-known nanocolumnar structure, which is endowed with high porosity (micro- and mesoporosity), as expected for thin films prepared by MS-OAD with axial substrate rotation during film growth.^29^ The *NiFe* and *NiFeOx* single-layer coatings presented a homogeneous aspect throughout their thickness. Meanwhile, a two-layer structure can be observed in the *NiFe/NiFeOx-200*, *NiFe/NiFeOx-500*, and *NiFe/NiFeOx-800* catalyst coatings, which is consistent with the growth of an oxide layer on top of a metallic layer. The equivalent thicknesses of each layer are gathered in Table 1.

**Table 1:** Equivalent Thickness of the Catalytic Layers Considered in This Work as Determined from the SEM Cross-Section Images in Figure S3 of Supporting Information 3^a^

	equivalent thickness (nm)		
catalyst coating	metallic layer	oxide layer	total
*NiFe*	800		800
*NiFeOx*		1100	1100
*NiFe/NiFeOx-200*	750	210	960
*NiFe/NiFeOx-500*	740	520	1260
*NiFe/NiFeOx-800*	740	710	1450

a Error bars are estimated in ±20 nm.

The XRD of the single metal layer or bilayer catalyst (Figure S4 in Supporting Information 4) depicted a series of peaks at 44.5°, 51.8°, and 76° that can be assigned to the diffractions of the (111), (200), and (220) planes of the fcc crystal structure of metallic nickel (JCPDS 01-087-0712)^31,41,42^ and at 37.0°, 42.9°, 62.2°, and 75° that can be assigned to the (111), (200), (220), and (311) planes of the fcc structure of NiO (JCPDS 01-078-0429).^43,44^ The shifts toward lower angular values of the measured diffraction peaks with respect to the corresponding JCPDS references observed for the oxide samples are consistent with an expansion of the crystal structure of this phase of about 1%, which we attribute to the random substitution of Ni ions by Fe ions within the fcc NiO network.^23^ On the other hand, from the Scherrer analysis of the metallic and oxide phases, crystallite sizes of 10–15 nm can be estimated for both samples (Table S1 in Supporting Information 4). These results suggest that iron atoms enter substitutionally into the nickel structure, forming either an alloy or a mixed NiFe oxide in the pristine state of the samples. Electrochemically (EC) treated samples depicted similar XRD diagrams, suggesting that the EC-modifications only affect the topmost surface of the deposited catalyst in contact with the solution and do not progress toward their interior. This result also shows that the EC-modified layer is much thinner than the thickness of the layered catalyst and that the induced physicochemical changes only affect the external surface of the deposited mesoporous structure.

The microstructure of the catalytic layers deposited on the stainless-steel plates revealed that the roughness of the plate substrates affects its morphology, which depicted a wrinkled structure where nanocolumns have been arranged into bundles, reproducing the grain boundaries of the stainless-steel plate surface (Figure S5 in Supporting Information 5). This morphology is a typical feature of MS-OAD thin films^45^ that preserves the formation of highly porous layers.^46^ Thus, , the catalyst coatings deposited on these plates depict a “cauliflower-bundle-like” mesoporous structure. This morphology was very similar for all the catalytic layers and bilayers prepared in this work, with a slightly higher prominence of the bundle structure in the bilayers, as expected for their higher thicknesses.^29^ A feature of this microstructure and its internal pore distribution is its high accessibility to the electrolyte, as confirmed by the electrochemical characterization of the catalysts reported in the following sections.

To obtain stable and homogeneous electrocatalytic layers, the catalyst deposited on the stainless-steel plates was conditioned using the cyclic voltammetry methodology described in the Methods section. The set of 200 voltammograms (Figure S6 in Supporting Information 6) depicted prominent cathodic and anodic features separated by ∼0.1 V, whose areas increase progressively through the conditioning process. This behavior is characteristic of a reversible electrooxidation reaction of Ni, which reaches saturation after a certain number of cycles.^23,37^ A comparison of the shape and intensity of the voltammogram features between the first, 20th, and last cycles of the conditioning process is included in Figure 1A,B, respectively, for *NiFe* and *NiFeOx* samples. On the other hand, Figure 1C includes a model-scheme for the evolution of cathodic and anodic features based on the description proposed by different authors, indicating the presence of Fe as dopant with the label *Fe.^47–51^

**Figure 1:**
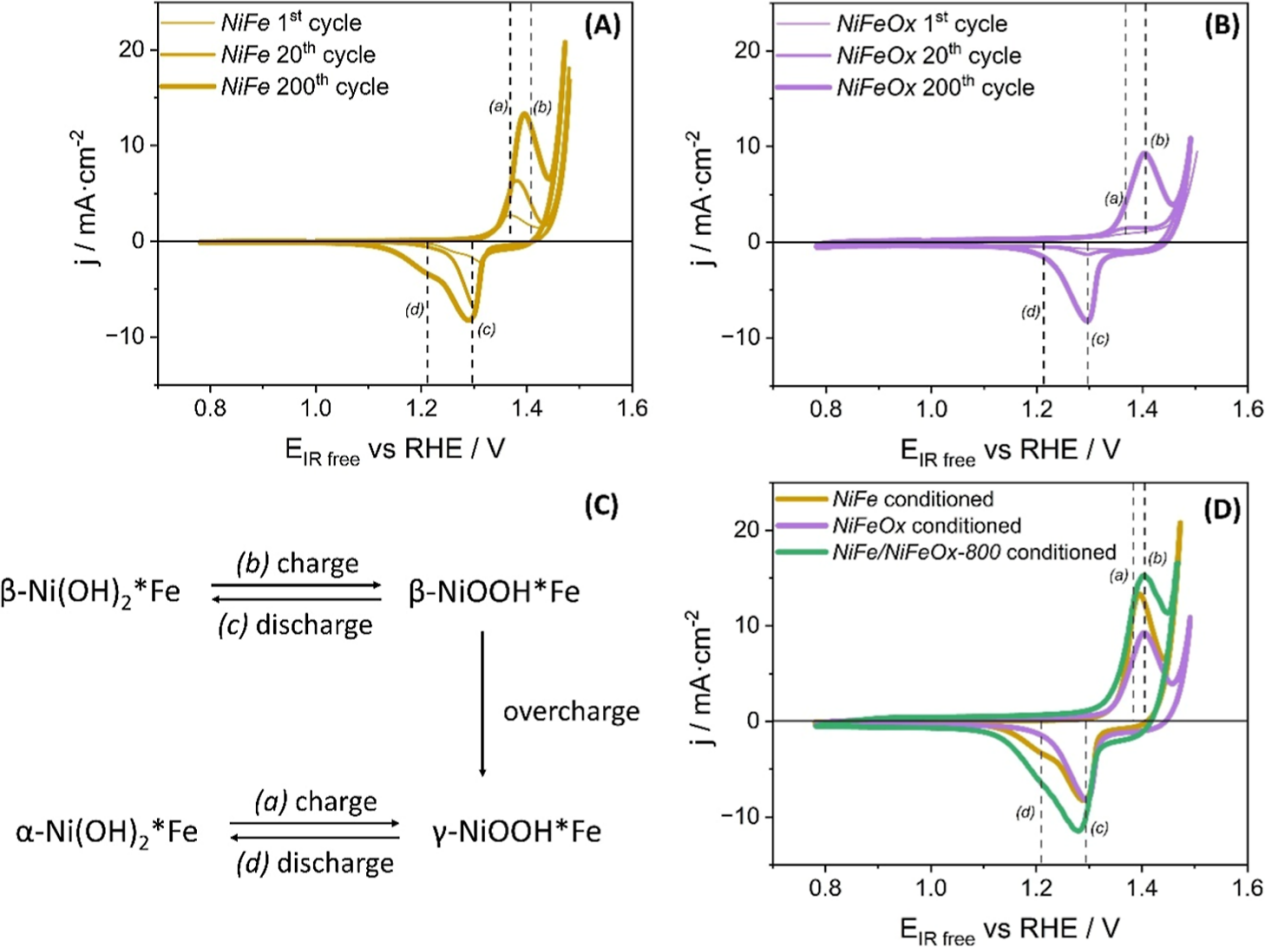
Comparison of the first, 20th, and last (i.e., 200) cycle anodic and cathodic currents of (A) NiFe and (B) NiFeOx samples acquired at 0.02 V/s in a N_2_ saturated 1 M KOH solution. (C) Traditional representation of the Bode scheme for the Ni redox transformation in alkaline media. (D) Comparison of the last (i.e., 200th) cycle of the conditioning procedure for *NiFe*, *NiFeOx*, and *NiFe/NiFeOx-800* catalysts acquired in the same conditions as (A,B).

Following the rationalization of Ni phases performed by Bode et al.^52^ in 1966, in the first cycle, the anodic current feature recorded in both samples at ∼1.38 V vs RHE is attributed to the formation of β-NiOOH*Fe from the oxidation of β-Ni­(OH)_2_*Fe species formed naturally in contact with KOH alkaline solution (process (*b*) in the scheme). This transition is supported by the appearance of a cathodic intense signal at ∼1.3 V in the return cycle, which corresponds with reversible formation of β-Ni­(OH)_2_*Fe from β-NiOOH*Fe species (process (*c*) in the scheme). When the first cycle of samples *NiFe* and *NiFeOx* is compared (Figure 1A,B), it is clear that the *NiFe* metallic layer exhibits peaks with a higher current intensity for the anodic and cathodic processes than *NiFeOx*. This difference arises from the presence of already oxidized Ni (and Fe) species in the catalyst layer, generated by the strongly oxidizing conditions during the reactive MS processing of *NiFeOx* layers. The experimental data indicate that after approximately 20 cycles, *NiFeOx* samples become activated, developing reversible and progressively more intense Ni^2+/3+^ anodic and cathodic peaks. Attending to the evolution of these anodic and cathodic current features with the CV cycles, the current maxima are shifted to higher and lower voltage values, respectively, indicating that the formation of β-NiOOH*Fe from β-Ni­(OH)_2_*Fe stabilizes, and the reversibility of the process decreases. Additionally, in the *NiFe* metallic layer, it is possible to determine one additional cathodic signal at ∼1.23 V vs RHE, which starts to be detectable around the 100th cycle, and it is associated with the electrochemical reduction of γ-NiOOH*Fe species to α-Ni­(OH)_2_*Fe (process (*d*) in the scheme). The recording of this cathodic signal also confirms the formation of γ-NiOOH*Fe species by an overcharge process, as indicated in Figure 1C. Analyzing the evolution of these signals across the cycles (further information in Figure S6 in Supporting Information 6), it is observed that in the *NiFe* metallic layer the cathodic current associated with process (*c*) increases over 150 cycles, indicating the formation of β species. From cycle 160 onward, cathodic current at 1.3 V vs RHE decreases while that at 1.23 V vs RHE increases, indicating the formation of α-Ni­(OH)_2_*Fe through process (*d*) after the overcharge process experienced by β-NiOOH*Fe. This behavior suggests that, after the conditioning procedure, in the metallic *NiFe* sample, Ni*Fe species are distributed in a mixed phase of α/γ and β/β hydroxides/(oxy)­hydroxides. On the contrary, the *NiFeOx* sample does not exhibit any evolution in the peak position over the CV cycles, showing the same trend during the conditioning procedure, where the β-NiOOH*Fe ↔ β-Ni­(OH)_2_*Fe are the main species formed through processes (*b*) and (*c*).

Comparing these results with those obtained for the bilayer catalyst (*NiFe/NiFeOx-800*) in the 200th cycle (Figure 1D), it is apparent that the resultant anodic and cathodic current in the bilayer sample depicts a mixed shape between those obtained from both monolayer catalysts, indicating that both layers (metallic at the bottom and oxides on top) are affected by the conditioning procedure.

The SEM images in Figure 2 recorded before and after the 200th CV cycles conditioning procedure, showcase that the surface morphology of the conditioned samples differs from that of the single-layer coatings of metal (*NiFe* sample) or the bilayer metal–oxide (e.g., *NiFe/NiFeOx 800*) catalysts. Thus, while as-prepared *NiFe* and *NiFe/NiFeOx-800* samples look rather similar (Figure 2A,D), after the electrochemical conditioning (Figure 2B,E) sample *NiFe* depicts a surface structure similar to that reported by Lin et al.^20^ for NiOOH-LDH*Fe platelets, suggesting that these structures are also formed during the electrochemical CV conditioning process of sample *NiFe*. Unlike this change in surface morphology in the cycled *NiFe* sample, the surface microstructure of the *NiFe/NiFeOx-800* sample was little affected by a similar conditioning treatment (Figure 2E). Differences increased after a CP aging test carried out for 12 h (see Methods section), after which *NiFe* sample (Figure 2C) appeared completely covered by the platelet structure while sample *NiFe/NiFeOx-800* (Figure 2F) presented an unmodified surface microstructure similar to that in its pristine state. This result clearly sustains a higher stability of the morphology of the oxide terminated metal–oxide bilayer catalyst samples.

**Figure 2:**
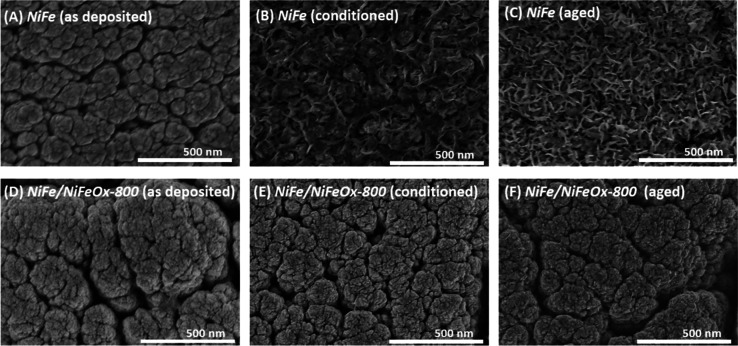
SEM images of NiFe (A–C) and NiFe/NiFeO-800 (D–F) catalyst samples deposited on stainless steel plates. A and D correspond to as-deposited samples, B and E correspond to conditioned samples, and C and F correspond to aged samples (see text).

Raman spectroscopy was used to assess the formation of oxide phases in the as-prepared, conditioned, and aged *NiFe*, *NiFeOx*, and *NiFe/NiFeOx-800* catalyst samples (cf. Figure 3). In the as-prepared state, clear differences are observed between the metallic *NiFe*- and the *NiFeOx*-containing samples. There is a main broad contribution in the NiFeOx and NiFe/NiFeOx-800 samples at ∼530 cm^–1^ (vertical red dashed line) with a shoulder at ∼480 cm^–1^ (vertical blue dashed line). These signals are consistent with the A_1g_ and E_g_ Raman active vibrational modes of Ni^2+^bonds,^53–56^ confirming the oxidic nature of these samples (detailed wavenumbers can be found in Table S2). Besides, there is a weaker broad band at ∼360 cm^–1^ (vertical black dashed line) which can be attributed in part to the initial substrate baseline (included in the graph as “bare plate”) and to a lesser extent to poorly crystallized iron oxide species,^57,58^ indicating the presence of Fe–O bonds for the *NiFeOx* and *NiFe/NiFeOx-800* samples. In the as-deposited *NiFe* sample, the intensity of bands between 450 and 600 cm^–1^ is very weak, indicating the small prevalence of oxide phases in the *NiFe* single-layer catalyst.^59^ After conditioning and aging treatments, the intensity of the band at ∼530 cm^–1^ of the *NiFe* sample increases significantly, confirming the formation of Ni–O species. However, the comparison between the shape of Raman spectra of the three samples reveals that the relative intensity of the oxide bands is much smaller for sample *NiFe*, indicating that even after a systematic usage of the *NiFe* catalyst as an oxidation electrode, it preserves an important metallic core, and the oxidation mainly affects the surface exposed to the electrolyte. The importance of the NiFe metallic phase to ensure a good electrical contact with the substrate will be analyzed in the next sections.

**Figure 3:**
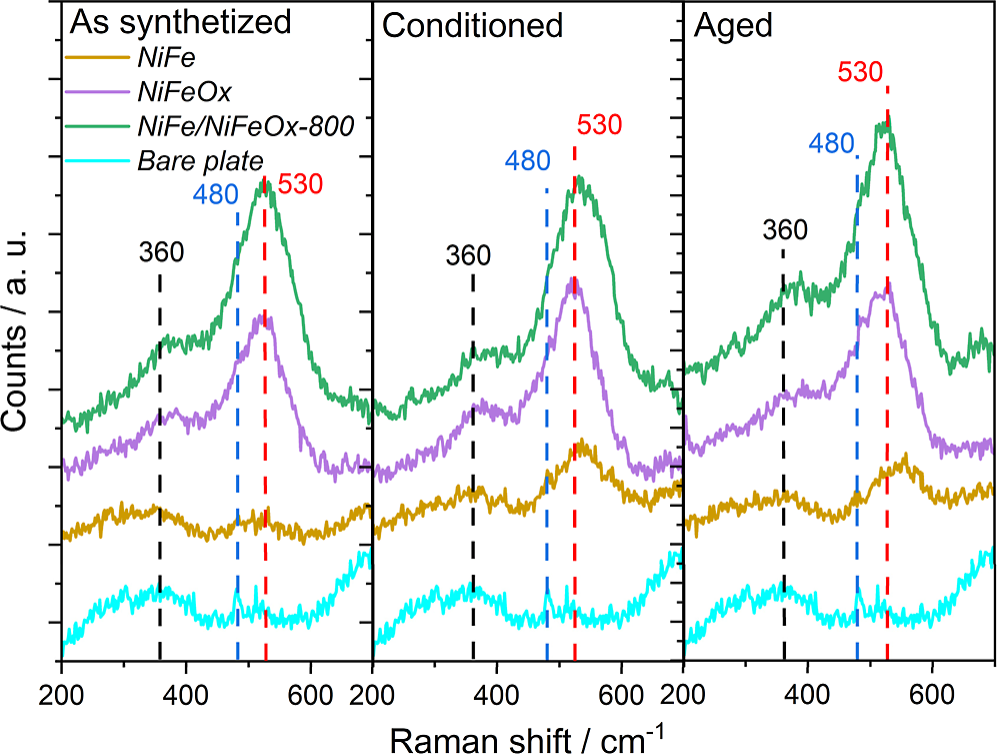
Raman spectra of as-deposited, conditioned, and aged *NiFe*, *NiFeOx*, and *NiFe/NiFeOx-800* catalyst samples compared with a “bare plate” sample. Typical vibrational bands of the different oxides and related compounds of the elements present in the samples are signaled in the figure with dashed lines. The black line represents a band attributable to Fe oxides, and the red and blue lines represent different Ni oxide species (specific wavenumbers reported in Table S2).

A further characterization of the oxide phase present in the catalysts was performed by FT-IR for oxide layers of variable thickness deposited on a silicon substrate. Spectra depicted a rise in the intensity of the FT-IR peaks (Figure S7 in Supporting Information 7) appearing in the 300–600 cm^–1^ wavenumber range, which can be associated with O–M bonds and depict a relative intensity proportional to the thickness of the oxide layer.^60,61^

Additional information about the chemical species present at the surface of the as-deposited, conditioned, and aged samples was obtained by XPS. The Ni 2p_3/2_ spectra of the *NiFe*, *NiFeOx*, and *NiFe/NiFeOx-800* samples as deposited are plotted in Figure 4A (see a more complete series of spectra in Figure S8 in Supporting Information 7). The spectra deconvolution performed using the B.E. values reported by Biesinger et al.^62,63^ for metal, oxides, hydroxides, and oxyhydroxides species (see Table S3 in Supporting Information 7) has allowed us to determine the distribution of Ni species at the surface of catalyst samples after the different electrochemical treatments. The results are reported in Figure 4B. Since the metallic Ni is highly affected by the atmospheric oxidation, the Ni 2p spectra for the *NiFe* sample depict a wide signal where it is possible to identify several oxidized and metallic Ni species.^64^ Thus, as-synthesized samples depict a spectral shape where the main species are Ni, NiO, and Ni­(OH)_2_. However, after the conditioning procedure, the surface composition of *NiFe* samples differs significantly from its initial state, reducing the relative concentration of Ni and NiO species and increasing that of Ni­(OH)_2_ and NiOOH species. In turn, for *NiFeOx* and *NiFe/NiFeOx-800* samples, surface changes were less pronounced after the conditioning procedure, which results in a small decrease in the NiO amount and a proportional increase of Ni­(OH)_2_ and NiOOH species. After the aging procedure, the trend changes and *NiFe* shows an increase in NiO and NiOOH species, while species Ni­(OH)_2_ decreases significantly. These phenomena are less pronounced in the *NiFeOx* sample, where the formation of Ni­(OH)_2_ and NiOOH species is lower when comparing the as-prepared and conditioned states. This behavior demonstrates the surface stability of this sample. In the as-prepared *NiFe/NiFeOx-800* bilayer catalyst, the amount of Ni­(OH)_2_ species is higher in the aged sample than in the conditioned sample, suggesting a possible synergistic effect between the metallic and oxidized NiFe layers, whereby the formation of Ni­(OH)_2_ from the metallic layer would depend on operation time.

**Figure 4:**
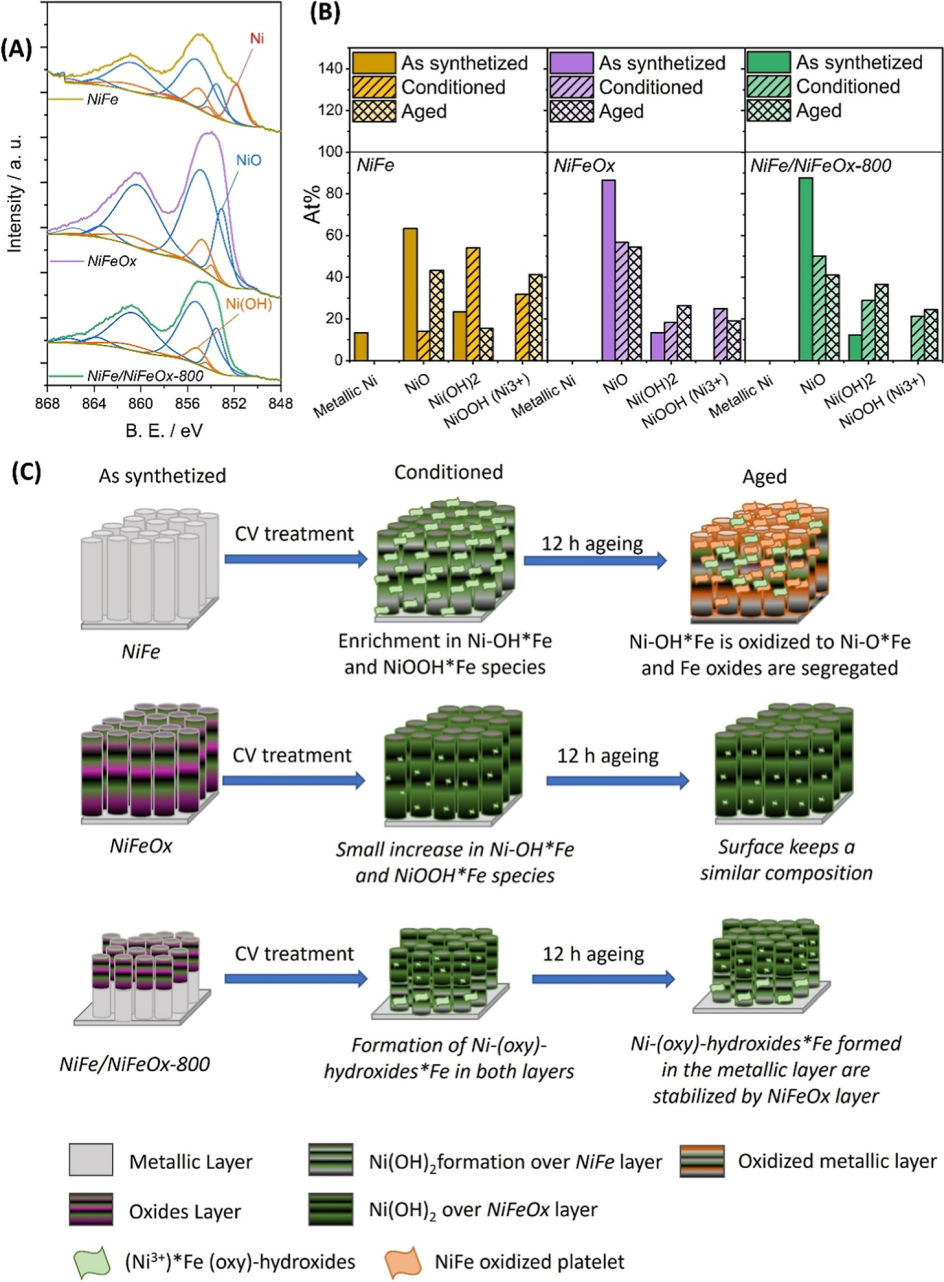
(A) Deconvoluted Ni 2p_3/2_ XPS spectra of as-prepared *NiFe*, *NiFeOx*, and *NiFe/NiFeOx-800* catalyst samples. (B) Columnar diagram with the surface distribution of nickel species calculated from the Ni deconvolution of Ni 2p_3/2_ spectra. (C) Schematic representation of surface evolution with the electrochemical treatments. *Surface oxidation due to exposure to ambient air has not been represented in the schematic, where only electrochemical transformations are highlighted.

A remarkable difference between samples is the higher relative amount of NiOOH species in the *NiFe* catalyst as compared with those found in the single-layer *NiFeOx* and bilayer catalysts. We can associate this difference with the observation by SEM of a platelet structure (Figure 2) in the single metal layer sample, supporting that upon conditioning and aging, they developed NiOOH*Fe lamellar structures on its surface.^65,66^

The distribution of Ni:Fe surface elemental concentrations as determined by XPS analysis of the *NiFe*, *NiFeOx*, and *NiFe/NiFeOx-800* catalyst samples revealed significant differences after various electrochemical treatments. In all cases, both as-prepared and conditioned samples show an equivalent Fe percentage of 10 at. %. However, after the aging procedure, in the *NiFe* sample, the Fe concentration increased significantly to 20 at. %, whereas in the *NiFeOx* samples, the Fe concentration did not vary substantially compared to this sample in its pristine state (13–14 at. %) (Table S4 in Supporting Information 7). A significant increase in the concentration of Fe in the *NiFe* sample was previously reported by Longo et al.,^34^ who, using “in situ” hard X-ray photoemission, detected a Fe segregation process during catalyst electrochemical usage. In the long term, this surface segregation of iron as Fe­(OH)_3_ species decreased the catalytic activity of the *NiFe* and therefore the overall capacity of these samples as effective OER catalysts.^34,67^ The herein reported XPS results indicate that such a segregation is hampered or significantly reduced in samples *NiFeOx* and *NiFe/NiFeOx-800*, an effect that must be attributed to incorporation during MS-OAD of an oxidized *NiFeOx* upper layer.

The previous considerations and proposed structural changes can be represented as in Figure 4C where a series of schemes for each sample describe their evolution from their pristine state to the situations after the conditioning process and after prolonged use (aging state). The main difference is the absence of a significant segregation of iron species in the single oxide and bilayer samples. It is to be remarked in this latter case that the oxide overlayer acts as a damping barrier, preventing the surface release of iron. On the other hand, the formation of platelet structures is prevented in these two samples, which must be considered more stable than the single-layer metallic catalyst.

### 3.2 Electrocatalytic Activity of Electrochemically Conditioned Catalysts Layers on SS Plates

#### 3.2.1 Effect of the Oxide Overlayer on the OER Activity of Bilayer Catalyst Coatings

The catalyst samples deposited on stainless-steel plates described in Table 1 were studied after the electrochemical conditioning process using a three-electrode cell in a 1.0 M KOH electrolyte solution (see the Methods section).

The activity toward the OER, obtained from LSV measurements, is shown in Figure 5A. Values of key electrokinetic parameters such as the Tafel slope and overpotential η vs *E*_1.23V vs RHE_^0^ at 10 mA·cm^–2^ (η_10_) derived from the LSV diagrams are shown in Figure 5B and Table 2. These data clearly prove that the composition and layered structure of the catalyst samples affect their electrochemical performance. Thus, the single-layer *NiFe* sample presents a lower overpotential than the single-layer oxide *NiFeOx* sample, while the Tafel slopes are quite similar in the two samples. We attribute the higher overpotential in the latter sample to its higher electrical resistance.^68,69^ Interestingly, the bilayer metal/oxide samples, with an equivalent thickness of the metallic layer similar to that of the single-layer *NiFe* sample, display similar overpotentials. It is also remarkable that the integration of a metallic layer and an oxide layer produces a significant decrease in the Tafel slope, a tendency that increases with the thickness of the *NiFeOx* top layer. This behavior suggests that additional catalytically active sites are formed in these bilayer catalysts.

**Figure 5:**
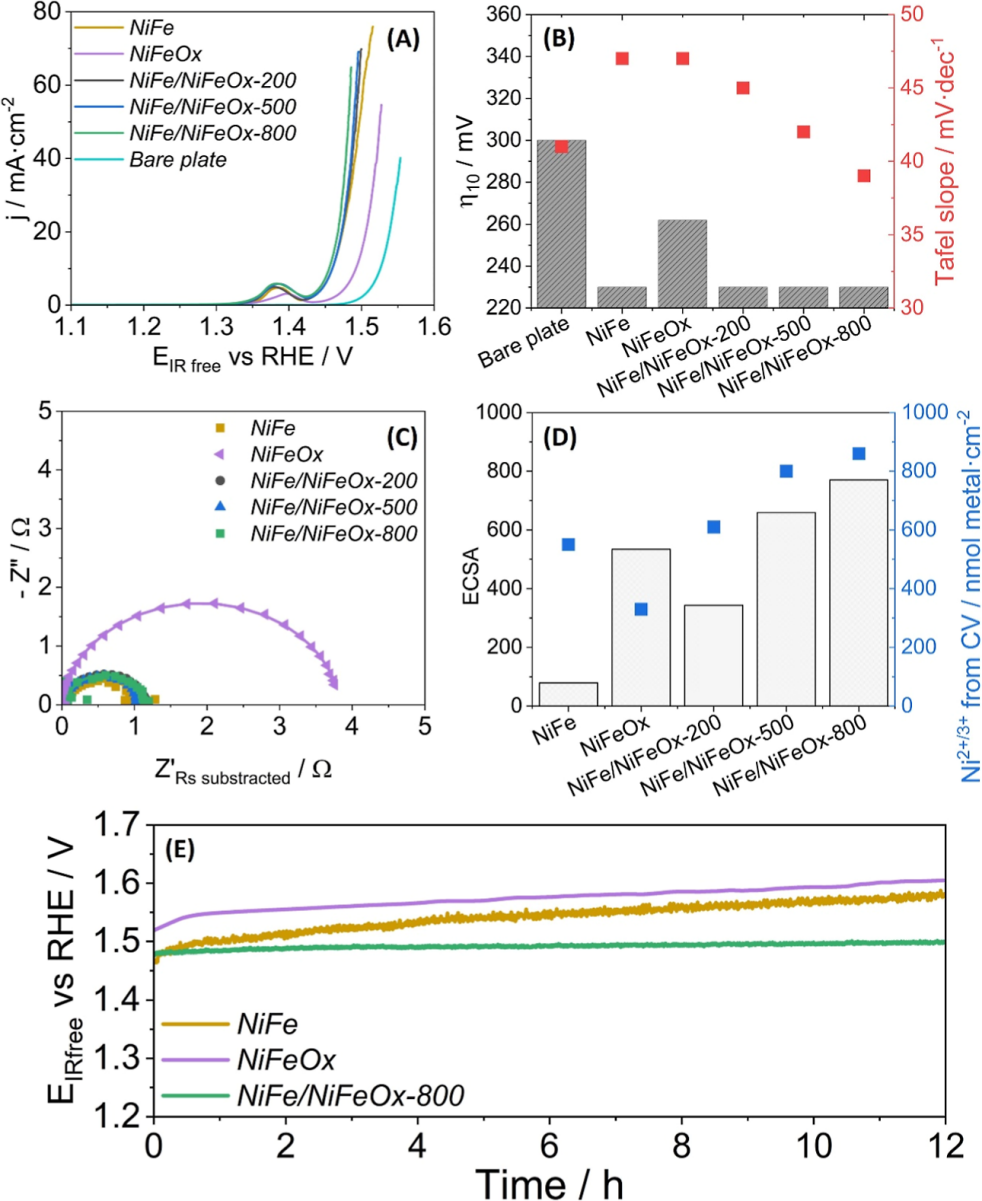
Electrochemical behavior of the conditioned catalyst samples. (A) LSV recorded at 0.005 V/s using a 1.0 M N_2_-saturated KOH as electrolyte. (B) η_10_ (bars) and the Tafel slopes (squares) determined from (A). (C) Nyquist plots acquired at 1.53 V vs RHE. (D) ECSA values (bars) and the amount of nickel oxide species formed during the conditioning process (blue squares). (E) Stability tests performed by CP at 0.025 A·cm^–2^ for 12 h.

**Table 2:** Electrokinetic Parameters Obtained for *NiFe*, *NiFeOx*, and *NiFe/NiFeOx-800* Samples after Conditioning and Ageing Procedures

	$\eta_{10mA\cdot {cm}^{-2}\;vs\;E=1.23V}$ (mV)		Tafel slope (mV·dec^–1^)		*R*_T_ (Ω) (*R*_T_ = *R*_1_ + *R*_2_)		stability test	
	conditioned	aged	conditioned	aged	conditioned	aged	η_25 average_ (mV)	ΔmV/h
*NiFe*	230	255	47	64	1.04	2.43	310	10
*NiFeOx*	262	284	47	57	3.86	6.72	340	7
*NiFe/NiFeOx-200*	230	244	45	48	1.17	1.46	263	2
*NiFe/NiFeOx-500*	230	240	42	45	1.06	1.39	255	2
*NiFe/NiFeOx-800*	230	228	39	42	1.14	1.27	260	2

To further investigate the electrochemical performance of the catalyst samples, they were characterized by EIS in a voltage range between 1.23 and 1.53 V vs RHE. In a preliminary analysis, a clear evolution with voltage was found in the Nyquist plots, which transformed from straight lines or very open circular curves to closed semicircles when approaching the voltage values at which the OER takes place (as an example, see in Figure S10 in Supporting Information 8, a series of plots taken for sample *NiFe/NiFeOx-800* at increasing voltages).

The most relevant results from the EIS analysis are gathered in Figure 5C, showing the Nyquist plots obtained at 1.53 V vs RHE (i.e., under the OER conditions) for the studied samples. These diagrams have been modeled using the equivalent circuit commonly employed to simulate water oxidation processes at anodes.^37,70^ This equivalent circuit (Figure S11, in Supporting Information 8) includes three resistance components, *R*_s_ linked to the electrolyte and *R*_1_ and *R*_2_ associated with the Ni^2+^ ↔ Ni^3+^ charge transfer and OER resistances, respectively, and two constant phase elements CPE1 and CPE2 associated with nonideal capacitive effects in these two processes. The fitting depicts a single semicircle, indicating that both the Ni-oxidation and the OER reactions have similar time constant values.^71^ The quality of the fitting was confirmed by evaluating the deviation between the experimental and simulated Bode plots.^47^

Fitting analysis reveals that the bilayer catalyst samples, including a metallic layer, present *R*_1_ and *R*_2_ resistance values much smaller than those found for the single-layer *NiFeOx* sample (Table S5 in Supporting Information 8). The total resistance (*R*_T_) (Table 2) of both processes (*R*_1_ + *R*_2_) for the bilayer catalyst was even smaller than the value found for the metallic single-layer *NiFe*, particularly after the aging treatment. These results stress some advantageous features of the bilayer catalyst. First, regarding the single oxide layer catalyst, the interface between the metal oxide catalyst and the metal catalyst layer in contact with the stainless-steel substrate helps to reduce the electrical resistance of charge transfer processes within the catalyst system and its support. Second, with respect to the single metallic catalyst, the oxide overlayer stabilizes the more reactive Ni­(OH)_2_ surface species, improving the charge transfer process and reducing the *R*_T_ values. Additionally, this stable Ni*Fe phases distribution minimizes the segregation of iron oxide moieties, which are deleterious for catalysis (see scheme in Figure 4C).

Another key parameter related with the electrochemical activity of electrodes is ECSA,^38,72^ which can be related to the double-layer capacitance (*C*_dl_) of the catalyst samples as deduced from CV measurements at non-Faradaic conditions and several scan rates (Figure S12 in Supporting Information 8).^73,74^ The ECSA values were obtained taking the substrate double-layer capacitance (*C*_S_) as a reference. The obtained results are shown in Figure 5D. This figure also displays a plot of the amount of charge, expressed in nmol per cm^2^ units, which is exchanged between Ni species and the electrolyte through the Ni^2+/3+^ electrochemical reaction.^73,75,76^ This parameter has been estimated from the area of the cathodic peaks at ∼1.23 and 1.3 V vs RHE after the conditioning procedure (Figure S13 in Supporting Information 8).^73,75,76^ As a first approximation, this magnitude can be taken as related to the number of reversible Ni centers involved in the OER. Interestingly, the results reported in Figure 5D graphs do not display a clear correlation between the number of electroactive species and the ECSA values. For example, *NiFeOx* sample has the lowest number of electroactive species, but its ECSA value is higher than those for *NiFe* or *NiFe/NiFeOx-200* samples. Conversely, the amount of electroactive species found in the bilayer catalyst samples is higher, a feature that might be accounted for by the sum of species at both the metallic and the oxide layers. This implies that the whole structure of the bilayer catalysts is accessible to the electrolyte, even for the thickest catalyst coatings. This feature ensures a relatively higher ECSA value for the oxidic layer which joined with the relatively higher concentration of electroactive species in the metal layer are factors contributing to the observed decrease in the Tafel slope values of the bilayer catalysts (cf., Figure 5B) and the enhanced OER catalytic activity found at higher current densities.^77,78^

Besides the electrochemical yield, the stability of the catalysts is crucial for their deployment in large-scale applications. Aging tests were carried out using a procedure that involved a CP at a current density of 25 mA·cm^–2^ for 12 h. Figure 5E shows the voltage evolution as a function of time for the *NiFe*, *NiFeOx*, and *NiFe/NiFeOx-800* samples. The lowest voltage increase after the 12 h test (approximately 1% rise) was found for the bilayer catalyst. To better characterize this evolution, LSV curves were recorded after the aging tests (Figure S14 in Supporting Information 8). η_10_ and Tafel slope parameters obtained from these plots, along with the *R*_T_ values determined by EIS for the samples subjected to conditioning and aging treatments, are summarized in Table 2. The values in this table show that η_10_ generally increased after aging, except for *NiFe/NiFeOx* samples. Tafel slopes, η_25_ and ΔmV/h followed a similar tendency to η_10_, where bilayer samples present the lowest values. Significantly, the *R*_T_ value after the aging treatment (Figure S15 in Supporting Information 8) also presented a minimum for these samples. These results suggest that the oxide overlayers contribute to improving the stability of the whole catalyst system, an effect that increases with the oxide layer thickness. The schemes in Figure 4C graphically describe the chemical and morphological processes, which likely contribute to the stabilization of the electrochemical behavior of sample *NiFe/NiFeOx-800*. The schemes in that figure highlight that the oxide/metal bilayer catalyst retards the formation of platelet structured NiFe-oxyhydroxides, as well as that of NiOx and FeOx species, which contribute to reducing the activity after the electrochemical aging. Another related effect is the increase in the number of active sites of the OER (cf., Figure 5D), which helps improving the bilayer’s overall catalytic efficiency and stability.

### 3.3 Single-Cell Studies and Scaling-Up Proof of Concept

Due to its exceptional electrocatalytic yield and stability, the *NiFe/NiFeOx-800* bilayer catalyst was deposited on the SS316L felt PTL (*PTL/NiFe/NiFeOx-800*) and tested as an anode in a membrane electrode assembly (MEA) realized in two prototypes of AEM electrolysis cells with electrodes of 5 cm^2^ and 64 cm^2^ geometrical area (see the photographs and scheme of this MEA cell in Figure S16 in Supporting Information 9). These proof-of-concept experiments were primarily carried out to demonstrate the possibility of using the MS-OAD procedure for the fabrication of catalysts onto the PTL for their integration into MEAs. An additional purpose was to compare the stabilities of these two anode catalysts in such a MEA configuration. It is noteworthy by these experiments that the use of a Ni coated PTL as cathode catalysts surely restricts the hydrogen production with respect to the amount expected for cathodes incorporating Pt-based catalysts.^79–81^

The electrochemical performance of the *NiFe/NiFeOx-800* catalyst coated PTL as an anode of the single cell was compared with that of a *NiFe* catalyst-coated PTL using the 5 cm^2^ cell area. Figure 6 showcases the morphological characteristics of these two as-deposited catalysts on the PTL substrates. It is worth pointint out that the catalysts do not cover completely the large voids within the fibrous structure of the PTL but form a coating conformally decorating the PTL fibers at the three/four more external levels from the surface. This is likely due to the MS-OAD characteristics of the deposition procedure, where atoms arrive from the gas phase to the substrate, and the equivalent thickness of the deposited catalyst layer (1–2 μm) is significantly less than the diameter (∼12 μm) of the fibers.

**Figure 6:**
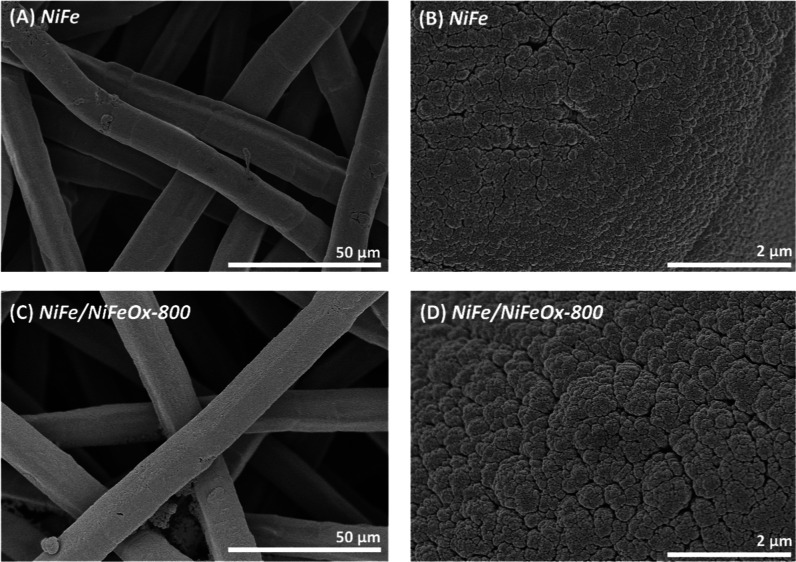
SEM pictures at different magnifications of (A,B) *NiFe* and (C,D) *NiFe/NiFeO-800* catalytic loads deposited over a SS316L PTL.

Polarization (*j*–*V*) curves in Figure 7A evidence that the *PTL/NiFe/NiFeOx-800* electrode requires a lower voltage (e.g., 3% less power consumption at 1.0 A/cm^2^) than the *PTL/NiFe* electrode to supply the same current. Besides this increase in efficiency, the cell demonstrated a higher stability when it was used for longer times. Stability was assessed following the evolution of the cell voltage in a CP aging test at a fixed current density of 0.6 A·cm^2^ for 45 h. The curves in Figure 7B show that after 45 h of operation, the voltage of the cell operating with the *PTL/NiFe/NiFeO-800* anode showed a slight degradation of 2.8 mV·h^–1^. However, this degradation is highly influenced by the behavior of the system in the first 5 hours. Considering the voltage rise for the last 40 h of the CP process, the value of the degradation rate decreases to 0.5 mV·h^–1^, one of the lowest increases reported in the literature for AEMWE cells.^82–85^ This voltage evolution was about one-third of that experienced by the cell with the *NiFe/PTL* anode. Furthermore, EIS analysis of the whole cell provided interesting clues to account for the differences in the complete cell resistance (*R*_Cell_) operated with each of these two catalysts. The Nyquist plots obtained at 1.8 V before and after the 45 h CP aging test (cf., Figure 7C) reveal an increase of the *R*_Cell_ values for the cell loaded with both catalyst samples, although this increase was higher for the cell loaded with the *NiFe* catalyst, where the *R*_Cell_ increase by a 100% after the stability test, while in the MEA loaded with the *NiFe/NiFeOx-800* catalyst, this increase was about 58% (values reported in Table S6 in Supporting Information 9). This is congruent with the considerations above about platelets’ decomposition and iron oxide segregation in the *FeNi* catalysts as schematized in Figure 4C. This would mean that changes in species distribution and microstructure occurring during prolonged electrochemical operation happen both when deposited onto a SS plate with the electrode immersed and conditioned in a three-electrodes cell or when the catalysts are deposited onto a PTL and the electrode is assembled with a membrane in a cell operating under alkaline conditions.

**Figure 7:**
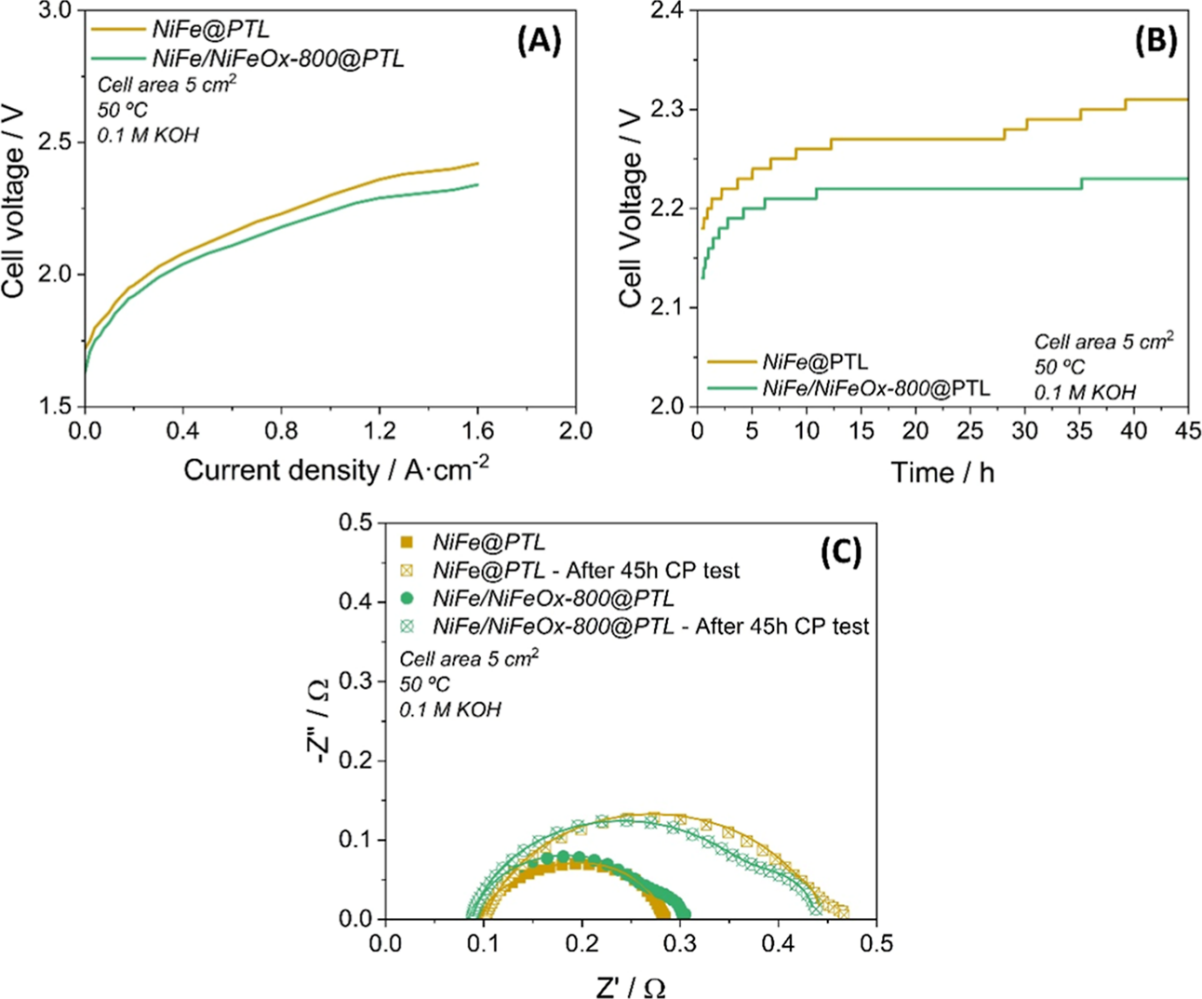
Electrochemical evaluation of *PTL/NiFe* and *PTL/NiFe/NiFeO-800* anodes integrated in a 5 cm^2^ single cell with an MEA configuration (0.1 M KOH electrolyte, Fumasep membrane, Ni catalyst-coated PTL cathodes). (A) *j*–*V* curves recorded from 0 to 1.6 A·cm^–2^. (B) Evolution of cell voltage during a CP test carried out at a fixed current of 0.6 A·cm^–2^ during 45 h. (C) Nyquist plots acquired at 1.8 V before and after the CP aging test in (B).

To further check the reliability of the *PTL/NiFe/NiFeOx-800* anodes and the possibility of proceeding with its preparation by MS-OAD in a large-area PTL, additional experiments were carried out using large-area (64 cm^2^) electrodes in a MEA configuration as used in commercial electrolyzers (Figure S16C in Supporting Information 9). It must be highlighted that the operation of the MEA with the MS-OAD catalysts developed in this work does not require the incorporation of ionomers, as is the case for conventional catalysts prepared by wet methods.^86,87^ Figure 8A shows the *j*–*V* curve recorded for upgraded electrodes under similar conditions to those previously employed in the 5 cm^2^ cell (i.e., 0.1 M KOH electrolyte, Fumasep FAA-3-50 membrane, Ni cathode catalyst deposited on the PTL, stainless steel felt PTLs and dry cathode mode). This figure depicts a shape and response (current densities of 0.6 and 1.0 A·cm^–2^ at 2.29 and 2.67 V, respectively) similar to that in Figure 7A for the small cell. However, the recorded voltage values are higher than those found in the 5 cm^2^ small cell. To explain this increase, it is necessary to take into account additional parameters, such as fluid/gas management, cell heating, electrode–membrane interfaces, etc., that generally have a negative impact on the cell performance as the electrodes area increases. The determined Faradaic efficiency for H_2_ production plotted in Figure 8B indicates that it increases at higher current densities, reaching values around 90%, which is a good mark for a laboratory test.^88^ For practical usage, electrolyzers must demonstrate reproducibility upon switch-off and switch-on operation. Figure 8C shows a series of voltage/current–time curves recorded with the aged catalyst for working periods of 5 h per day during 5 days, applying a maximum value of 0.6 A·cm^–2^ of current density and a cutoff polarization value of the cell of 2.2 V. The figure shows that for all switching events, the cell rapidly reaches the cutoff value, and the current density stabilizes at around 0.3–0.4 A·cm^–2^. The current profile recorded among the first 3 days suggests that the system experiences an activation process, reaching its maximum stable value in the fourth day of work. This short-term experiment supports that the bilayer catalyst behavior remains substantially stable, also on these large-area PTLs. Moreover, they demonstrate a rather robust and reproducible behavior as required for large-area industrial applications. Further work is in progress to optimize the performance of these MEA-based processes, characterize the PTL deposited catalysts, and optimize both the anodic and cathodic reactions in the quest for Pt-free electrolysis cells and stacks working with MS-OAD catalysts.

**Figure 8:**
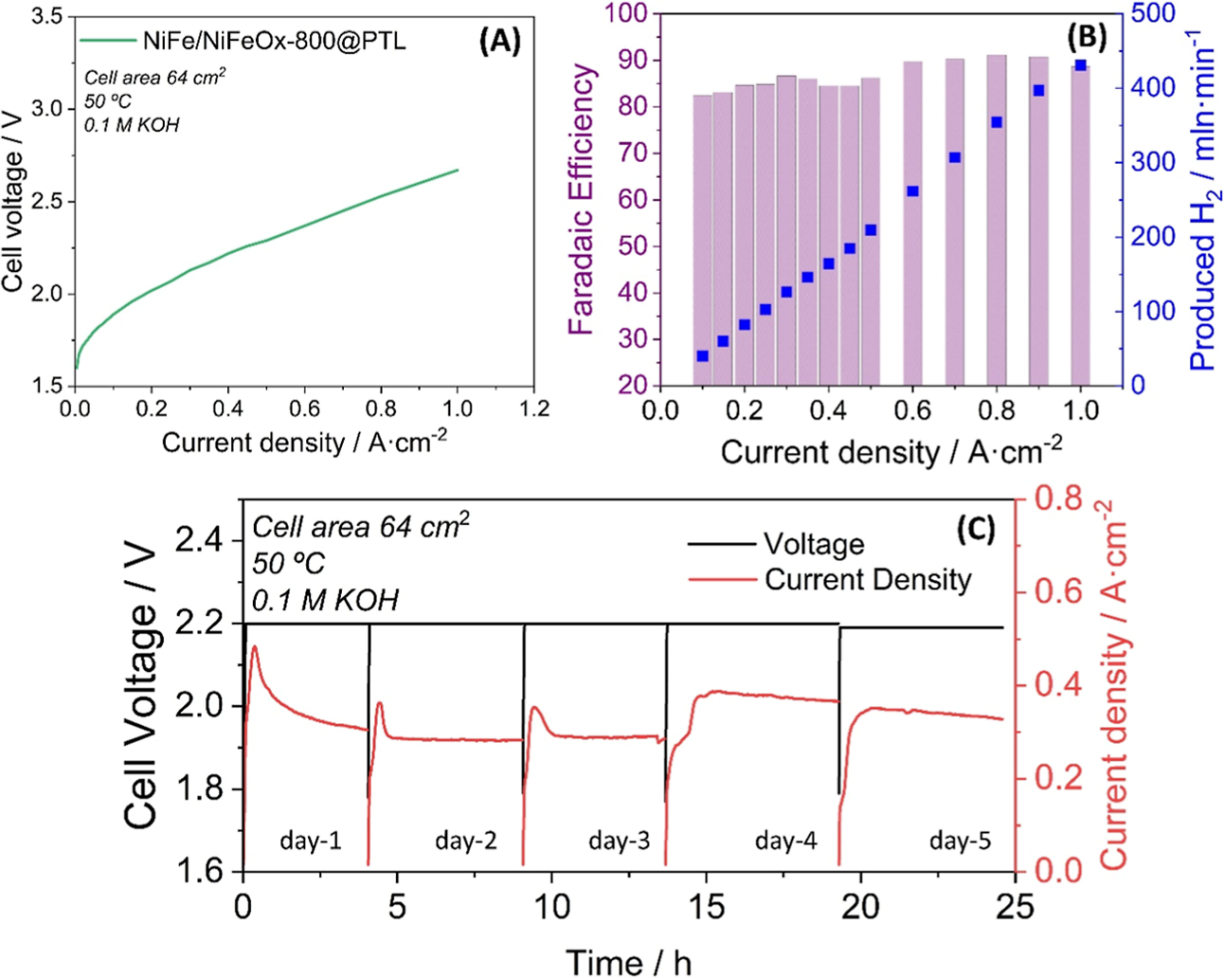
Selected electrochemical tests carried out with the single cell of 64 cm^2^ electrode area prototype. (A) Voltage vs current density. (B) Faradaic efficiency (bars) and hydrogen production yield (squares) vs current density. (C) Series of voltage vs time curves recorded for working periods of ∼5 h per day during 5 days, upon polarization of the cell at 2.2 V.

## 4 Conclusions

In this work, we have confirmed the usefulness of the MS-OAD technique for the fabrication of electrocatalysts for the OER. In a leap forward from previous results with this technique, we have proved here that this procedure can be applied for the synthesis of metal–oxide bilayer catalysts. The chemical composition selected for this work has been the common NiFe (10% Fe) formulation known for its high electrochemical activity as an anode for water splitting. A second aspect addressed in this work is the fabrication of large-area electrodes for their implementation in single cells with a MEA configuration. In this regard, the proof of concept presented as the final part of this paper demonstrates that bilayer catalysts can be efficiently deposited by MS-OAD on the large-area PTL substrates commonly used for the industrial manufacturing of AEMWE cells and stacks. Moreover, the ionomer-free operation for a long time of large-area membrane cells provided with the bilayer catalysts has proven the possibilities of the manufacturing process for the fabrication of devices with sizes relevant for industrial applications.

Besides these application-oriented conclusions, the paper provides key results of fundamental character that advance the knowledge of the chemistry of FeNi and FeNiOx catalysts and justify that bilayer catalysts present a higher activity and a longer stability in comparison with single-layer oxide and metallic catalysts. Interestingly, both features were confirmed when working in a three-electrode cell and, to a lesser extent, in the MEA device. Several factors have been identified as contributing to the higher performance of the bilayer catalysts. These factors synergistically combined in the bilayer catalysts provide, among other benefits, improved electrical contact with the substrate, a stabilizing effect over the external oxyhydroxide phase formed during electrocatalyst conditioning, and the development of a porous microstructure that provides a higher electrochemically active area and a higher number of active catalytic sites. Evidence of these effects has been obtained thanks to a precise characterization of the catalysts at different stages of their usage protocol: as prepared by MS-OAD on SS plates, after conditioning by electrochemical cycling and after an aging process for relatively long times. This thorough characterization has been accompanied by a precise electrochemical analysis of reaction parameters and their differences and evolution along this study. We deem that the results of this work support the advantages provided by ionomer-free thin film catalyst coatings prepared by MS-OAD where different layers with specific formulations and microstructures are stacked in a continuous manner to ensure multifunctionality and higher activity for specific reactions.

## Supplementary Material



## Notes

Further information about calculations, characterization procedures, electrochemical methods, and additional graphs of results (PDF)
