# Supplementary material for: Ionomer-Free NiFe/NiFeO Bilayer Oxygen Evolution Reaction Electrocatalyst Prepared by a Magnetron Sputtering at Oblique Angle Bottom-Up Deposition Method
Source: ACS Catal. 2025 Oct 17;15(21):18123–37. doi: 10.1021/acscatal.5c04915 (PMC12606170; doi:10.1021/acscatal.5c04915)
Supplement: Supplementary file 1 [file cs5c04915_si_001.pdf]

Supporting information

Ionomer-free NiFe/NiFeO bilayer oxygen evolution reaction electrocatalyst prepared by a magnetron sputtering at oblique angle bottom-up deposition method

Authors

José Manuel Luque-Centeno[a,*], Álvaro Carmo-Delcán[a], Mikel Martínez-Olaizola[a], Celia Gómez-Sacedón[b], Antonio de Lucas-Consuegra[b], Agustín R. González-Elipe[a], Francisco Yubero[a], José Javier Brey Sánchez[c,d], Jorge Gil-Rostra[a,*].

[a] *Laboratory of Nanotechnology on Surfaces and Plasma, Institute of Materials Science of Seville (CSIC - Univ. Sevilla), Av. Américo Vespucio 49, E-41092, Sevilla, Spain.*

[b] *Department of Chemical Engineering, School of Chemical Sciences and Technologies, University of Castilla-La Mancha, Avda. Camilo José Cela 12, E-13071, Ciudad Real, Spain.*

[c]*H2B2 Electrolysis Technologies, Moriscas 46-48, Pol. Ind. La Isla. Dos Hermanas, E-41703, Sevilla, Spain.*

[d]*Department of Engineering, Universidad Loyola Andalucía, Avenida de Las Universidades s/n, Dos Hermanas, E-41704, Sevilla, Spain.*

* Corresponding authors: jmanuel.luque@icmse.csic.es ; jorge.gil@icmse.csic.es

Keywords: Bilayer Electrocatalysts; Magnetron Sputtering; NiFe Catalysts; Anion Exchange Membrane Water Electrolysis; Oxygen Evolution Reaction; Layered Double Hydroxide; Hydrogen.

Supporting information SI1.- Morphological characterization of the SS and PTL substrates and effect of cleaning procedure

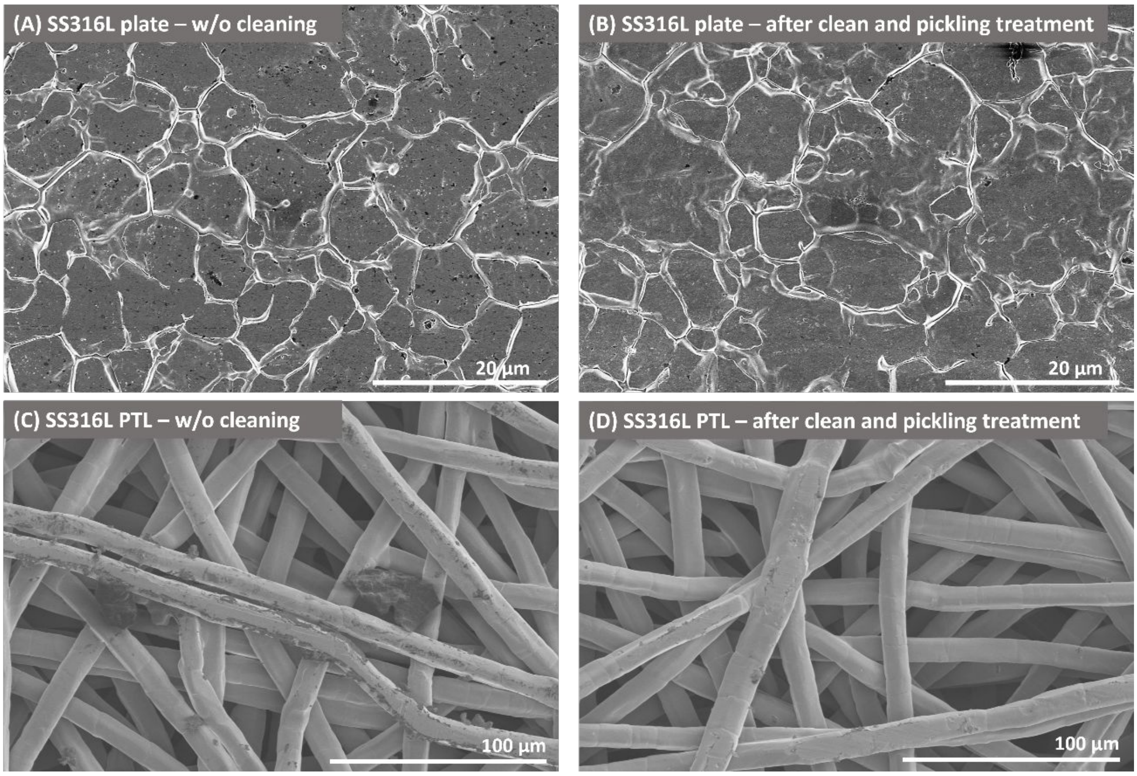

***Figure S1**. SEM images of SS316L substrates used as catalyst supports. Plates before (A) and after (B) cleaning and pickling treatment. Felts before (C) and after (D) cleaning and pickling treatment*

Supporting information SI2.- Three electrodes cell and area selected for electrochemical analysis onto the SS plates.

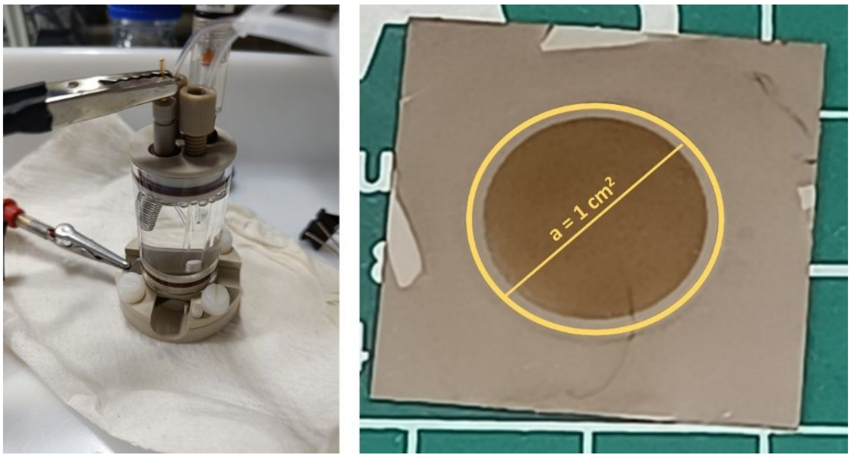

***Figure S2.** Left: electrochemical three-electrode system. Right: SS plate covered with catalyst after electrochemical test. The dark brown area corresponds to the geometrical area selected for analysis.*

Supporting information SI3.-Microstructure and equivalent thickness of catalyst coatings deposited on a flat silicon substrate.

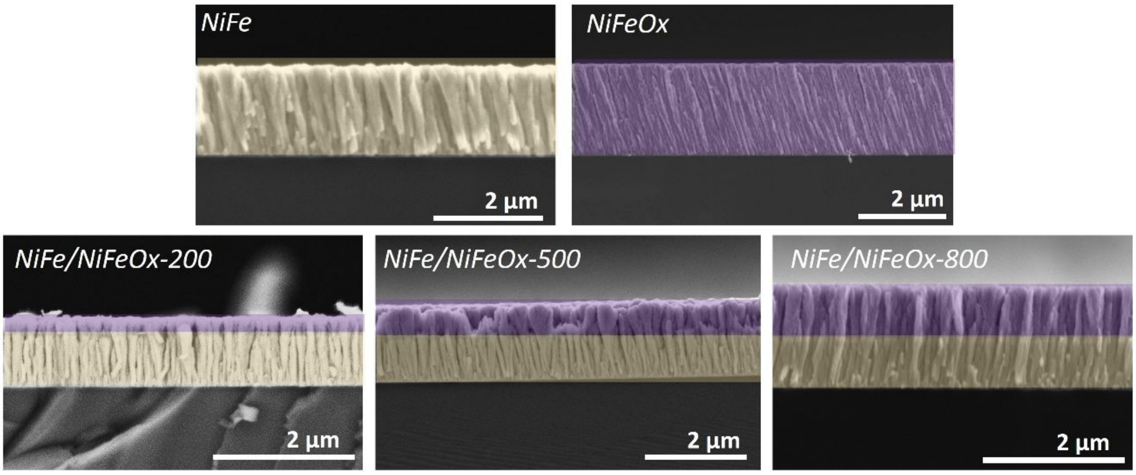

Figure S3. *SEM cross sections of catalyst films deposited on control polished silicon wafer pieces considered in this work. Equivalent thicknesses of the catalyst loads (NiFe layers are highlighted in pale yellow and NiFeOx layers in purple colour) are obtained from these images.*

Supporting information SI4.- XRD characterization of catalyst coatings

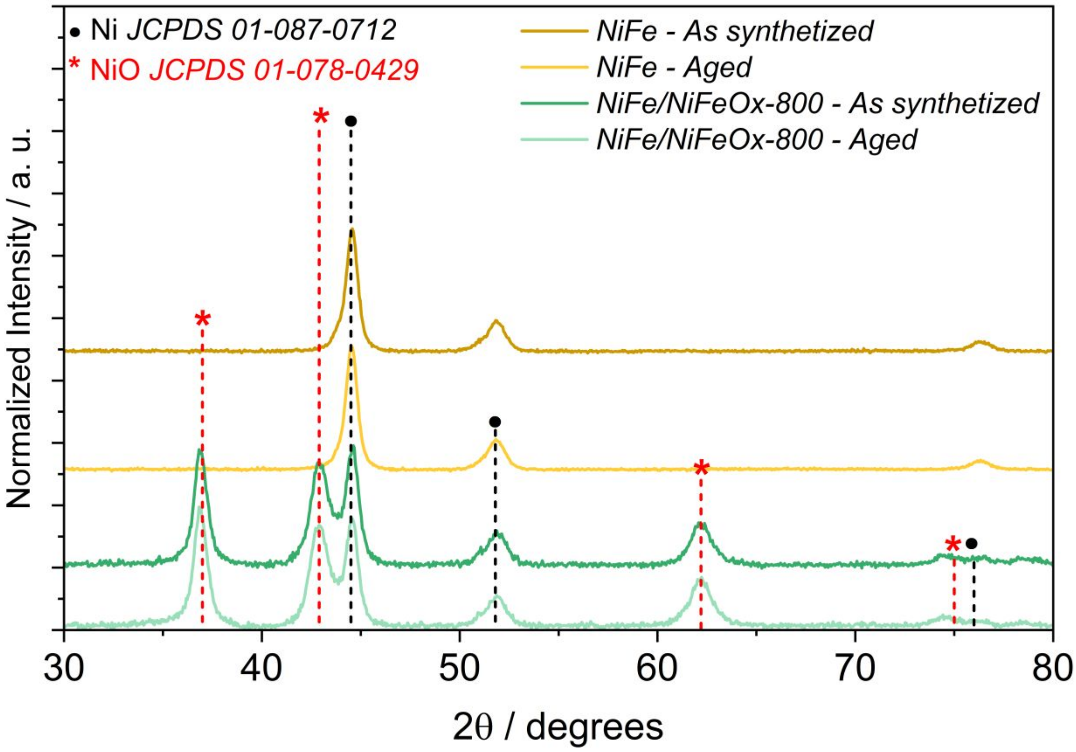

Figure S4. *XRD patterns obtained for as synthetized and aged NiFe (yellow lines) and NiFe/NiFeOx-800 (green lines) catalytic layers.*

The crystallite sizes of the different samples have been calculated using the Scherrer equation (**eq. S1**) from the Ni (200) and the NiO (220) diffractions and reported in **table S1**. All the samples present similar domains sizes between 10 and 15 nm.

***Table S1**. Crystallite sizes calculated from **figure S4**. Errorbars to be considered of +/- 1 nm*

Sample	*L_c Ni (2 0 0)*	*L_c NiO (2 2 0)*
NiFe As synthetized	11 nm	-
NiFe Aged	13 nm	-
NiFe/NiFeOx-800 As synthetized	15 nm	12 nm
NiFe/NiFeOx-800 Aged	10 nm	11 nm

For these calculations, Scherrer equation has been employed considering *K* as a constant, λ the wavelength of x-rays employed, the Full Width at Half Maximum (FWHM).

$$L_C = \frac{K \cdot \lambda}{FWHM \cdot cos\theta} \qquad \textbf{eq. S1}$$

Supporting information SI5.- Microstructure analysis of catalysts deposited onto SS316L metallic plates as electrode support.

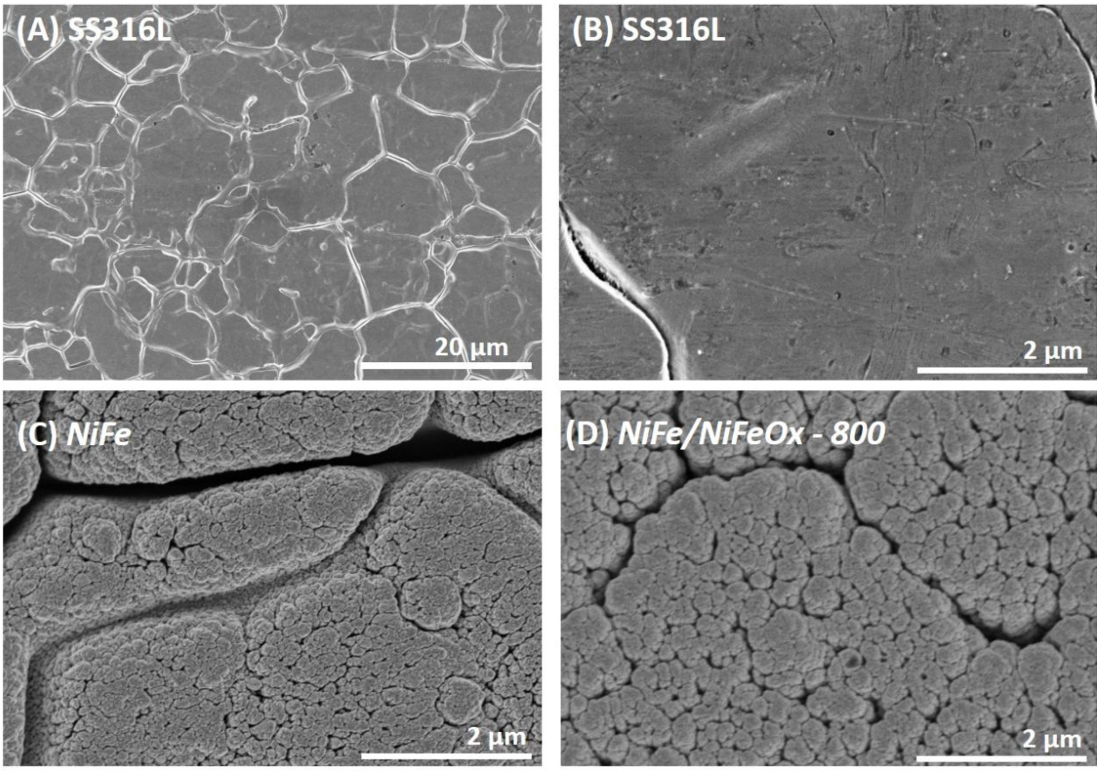

***Figure S5**. SEM images of pristine SS plates at two magnifications (A) and (B) and the same plate after deposit of NiFe (C) and NiFe/NiFeOx-800 (D) catalyst loads.*

Figure S5 shows the effect of roughness features of the SS316L plate on the catalysts layer morphology. It shows SEM images of pristine SS plates at two magnifications (A) and (B) and the same plate after deposit of *NiFe* (C) and *NiFe/NiFeOx-800* (D) catalyst loads. It is apparent that the catalyst morphology follows that defined by the plate surface grains, while still depicting an open and porous microstructure, particularly pronounced for the bilayer sample.

Supporting information SI6.- Electrochemical conditioning of samples using a cyclic voltammetry (CV) procedure.

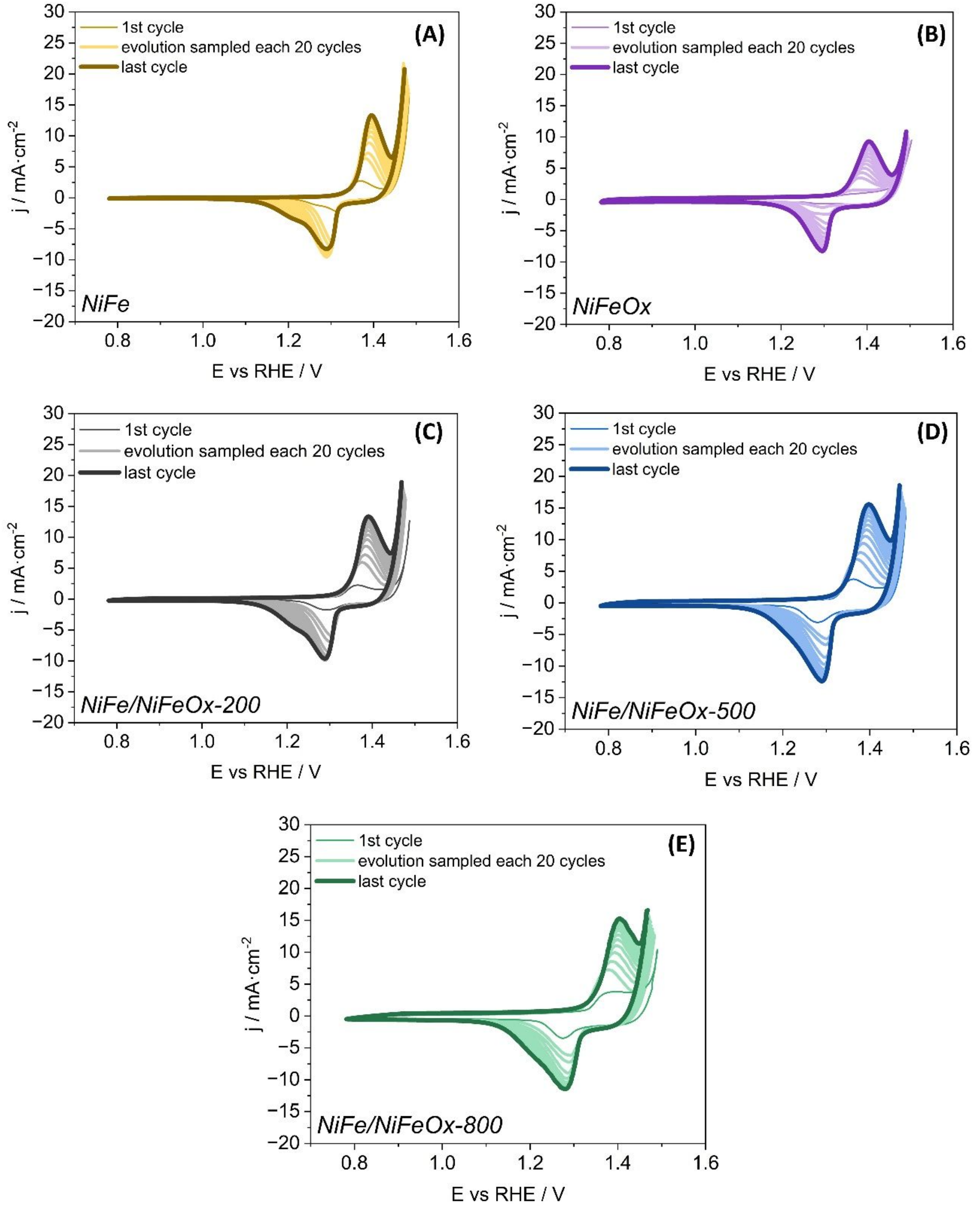

Figure S6. *Conditioning of (A)NiFe, (B) NiFeOx, (C) NiFe/NiFeOx-200, (D) NiFe/NiFeOx-500, and (E) NiFe/NiFeOx-800 catalyst samples deposited on SS plates by cyclic voltammetry (200 cycles). Experiments performed in 1M KOH N_2-saturated solution at 0.02 V/s scan rate. The x-axis corresponds to voltage values corrected by the voltage rise due to the electrolyte's resistance.*

Note in these experiments the progressive evolution in intensity and shape of the anodic and cathodic features with the number of cycles. This evolution sustains a progressive increase in the number of reversible species of nickel upon the electrochemical cycling of the catalyst coatings.

Supporting information SI7.- Surface characterization by Raman, FT-IR and XPS.

Table S2*. Raman wavenumbers employed in* ***Figure 3****.*

Specie	*Wavenumber (cm^{-1})*	*Mode*
Fe-O	~360	T_1
Ni^{2+}-O	520	A_{1g}
Ni^{2+}-O	470	E_g

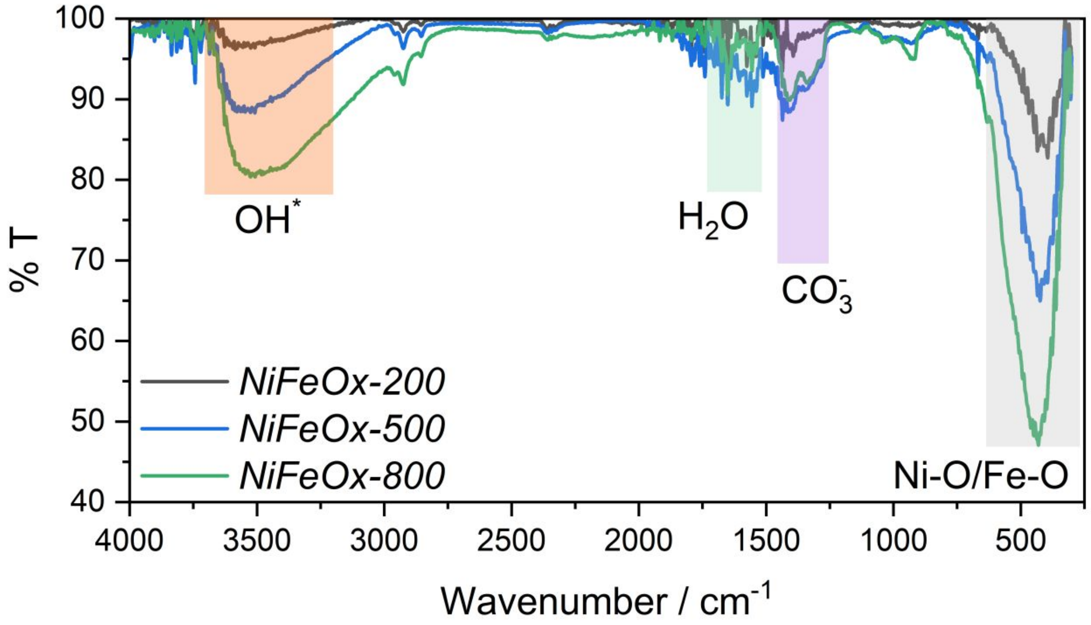

Figure S7. *Transmission FTIR spectra of NiFeOx-200, NiFeOx-500 and NiFeOx-800 oxide layers deposited on intrinsic polished silicon wafer pieces.*

Figure S7 shows transmission FTIR spectra of the NiFeOx oxide layers as pattern of the second layer deposited in the bilayer *NiFe/NiFeOx-200*, *NiFe/NiFeOx-500* and *NiFe/NiFeOx-800* in as synthetized samples. In all of them, four well-defined groups of bands can be identified. Two bands around 3000 – 3600 cm^{-1} and 1500 – 1800 cm^{-1} are associated with the O—H* stretching vibrations in the brucite-like layers of adsorbed humidity. Note that the intensity of these bands increases with the oxide layer thickness, indicating a progressively larger amount of OH^- species. This can be attributed to the formation of a thicker NiFe hydroxides layer for the sample with the thicker oxide (i.e. *NiFe/NiFeOx-800*) or an increase in the amount of adsorbed water. The 1350-1250 cm^{-1} band belongs to the presence of CO_3^{2-} intercalated anion vibration. Finally, the band, around 400 cm^{-1}, is related to the stretching vibrations of Ni—O and Fe—O, which is also attributed to the formation of Ni and Fe oxidized species.

According with the previous studies reported by Biesinger et al. Ni $2p_{3/2}$, Fe $2p_{3/2}$ and O 1s high resolution orbitals have been deconvoluted using the following binding energies for the reported bonds:

Table S3. XPS binding energies employed for spectral fitting.

**Compound**	**Peak 1** eV	**Peak 2** eV	**Peak 3** eV	**Peak 4** eV	**Peak 5** eV	**Peak 6** eV	**Peak 7** eV
Ni $2p_{3/2}$ orbital							
Ni metal	852.6	856.3	858.7				
NiO	853.7	855.4	860.9	864.0	866.3		
$Ni(OH)_2$	854.9	855.7	857.7	860.5	861.5	866.5	
NiOOH	854.6	855.3	855.7	856.5	857.8	861.0	864.4
Fe $2p_{3/2}$ orbital							
Fe metal	706.6						
FeO	708.4	709.7	710.9	712.1	715.4		
Fe(3+)	710.0	711.0	711.9	713.0	714.1	719.5	
FeOOH	710.3	711.3	712.2	713.3	714.4	719.7	
O 1s orbital							
Ni-OH	530.9						
Ni-O	529.3						
Fe-OH	529.9						
Fe-O	530.1						
H_2O abs	532.2						

The deconvolution of Ni $2p_{3/2}$ high resolution spectra among the different electrochemical treatments is depicted in the **figure S8**:

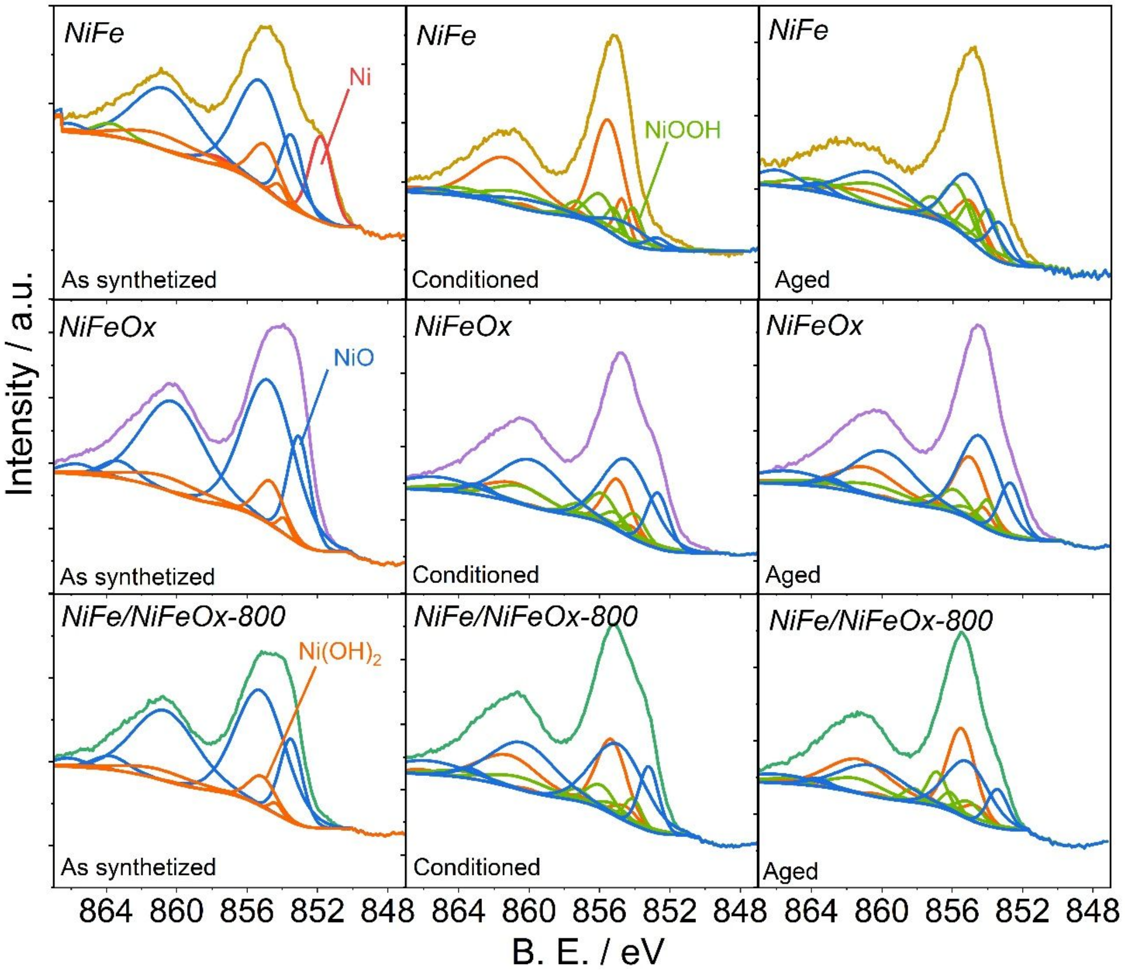

Figure S8. Example of XPS deconvolutions on Ni $2p_{3/2}$ high resolution spectra.

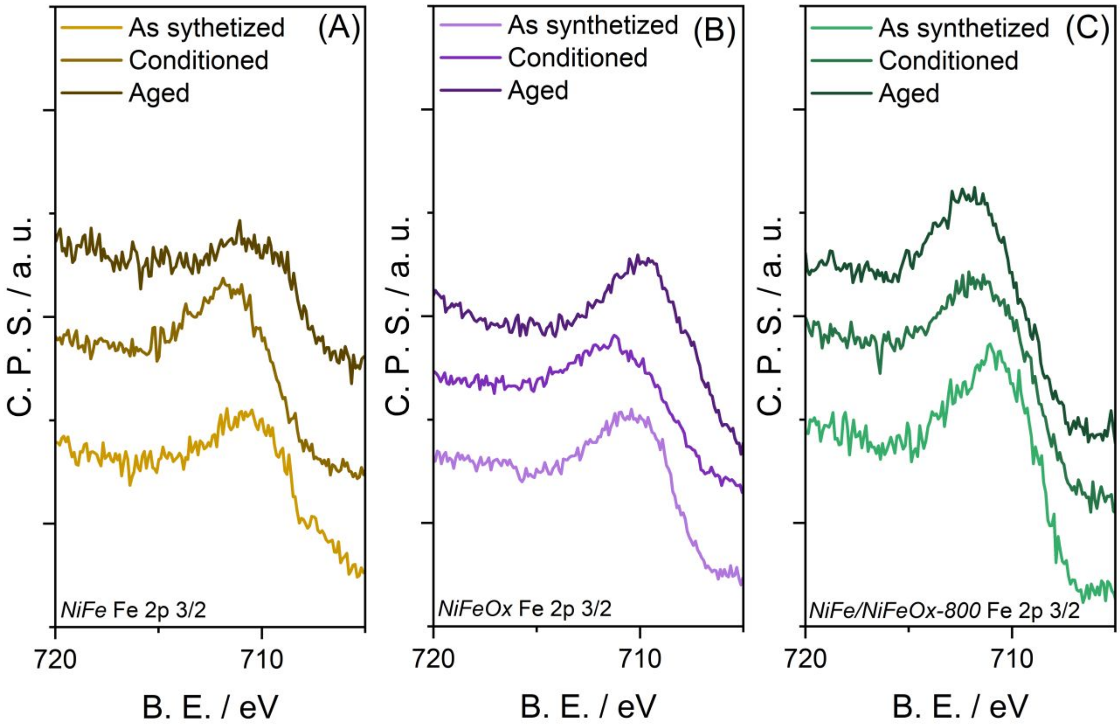

Figure S9. Fe $2p_{3/2}$ high resolution spectra of the as synthetized, conditioned and aged *NiFe*, *NiFeOx* and *NiFe/NiFeOx-800* samples.

Table S4*. Ni/Fe relative atomic percentages at the surface of samples calculated from the relative intensity of Ni $2p_{3/2}$, Fe $2p_{3/2}$ high resolution XPS peaks.*

Sample	**Ni At%**	**Fe At%**
As synthetized		
NiFe	87	13
NiFeOx	89	11
NiFe/NiFeOx-800	89	11
Conditioned		
NiFe	89	11
NiFeOx	89	11
NiFe/NiFeOx-800	89	11
Aged		
NiFe	80	20
NiFeOx	87	13
NiFe/NiFeOx-800	86	14

Supporting information SI8.- Electrochemical characterization of samples in a three-electrodes half-cell.

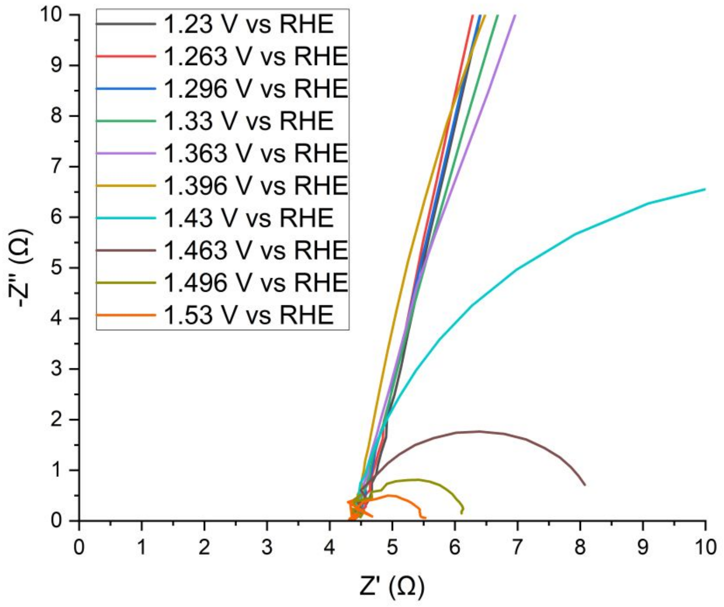

***Figure S10**. Nyquist plots obtained for the NiFe/NiFeOx-800 sample, recorded at increasing voltages.*

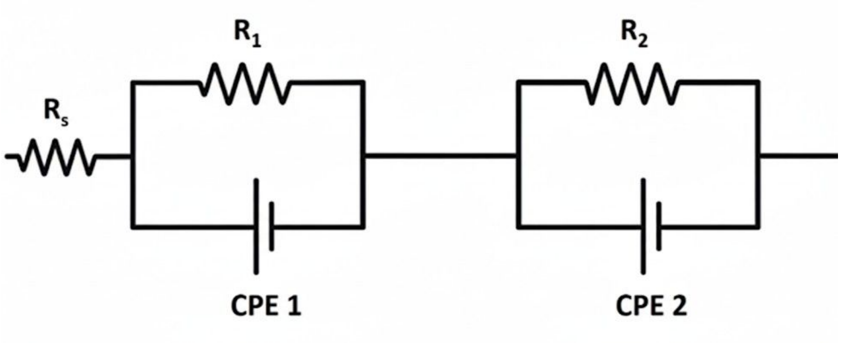

***Figure S11**. Equivalent circuit employed to perform EIS fitting analysis of Nyquist plots*

EIS spectra have been fitted using Zview software from Scribner® using the equivalent circuit plotted in **Figure S11**, where R_s is the resistance due to the solution, R_1 is the charge transfer resistance due to the Ni^{2+}/Ni^{3+} oxidation process and CPE 1 its constant phase element, and R_2 and CPE 2 are the charge transfer resistance and constant phase element for the oxygen evolution reaction process. **Table S5** gathers the values of R_1 and R_2 resistances determined by this fitting analysis of the different samples after their conditioning and ageing treatments.

Table S5. *Resistance values determined from the analysis of Nyquist plots acquired in half cell configuration reported in* ***Figure 5C*** *and equivalent EIS plots recorded after ageing treatments.*

	After conditioning (from figure 5 – C)		After aging	
**catalyst sample**	**R_1 (Ohm)**	**R_2 (Ohm)**	**R_1 (Ohm)**	**R_2 (Ohm)**
NiFe	0.11	0.93	0.14	2.29
NiFeOx	0.12	3.74	0.20	6.52
NiFe/NiFeOx-200	0.02	1.15	0.02	1.45
NiFe/NiFeOx-500	0.06	1.00	0.01	1.38
NiFe/NiFeOx-800	0.05	1.09	0.09	1.18

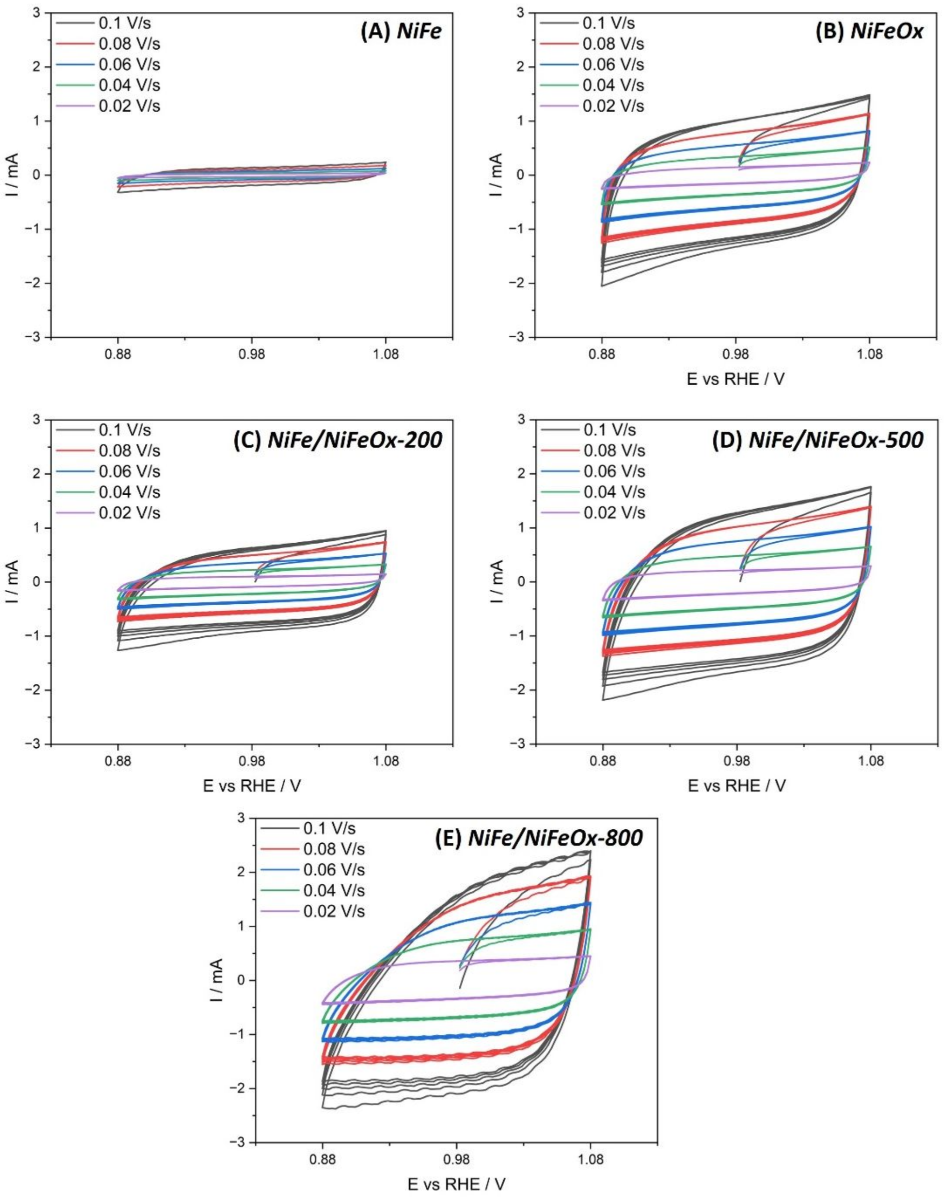

Figure S12. *CV measurements registered at non-faradaic conditions in 1.0 M KOH N_2-saturated solution and several scan rates of (A) NiFe, (B) NiFeOx, (C) NiFe/NiFeOx-200, (D) NiFe/NiFeOx-500, and (E) NiFe/NiFeOx-800 catalyst samples.*

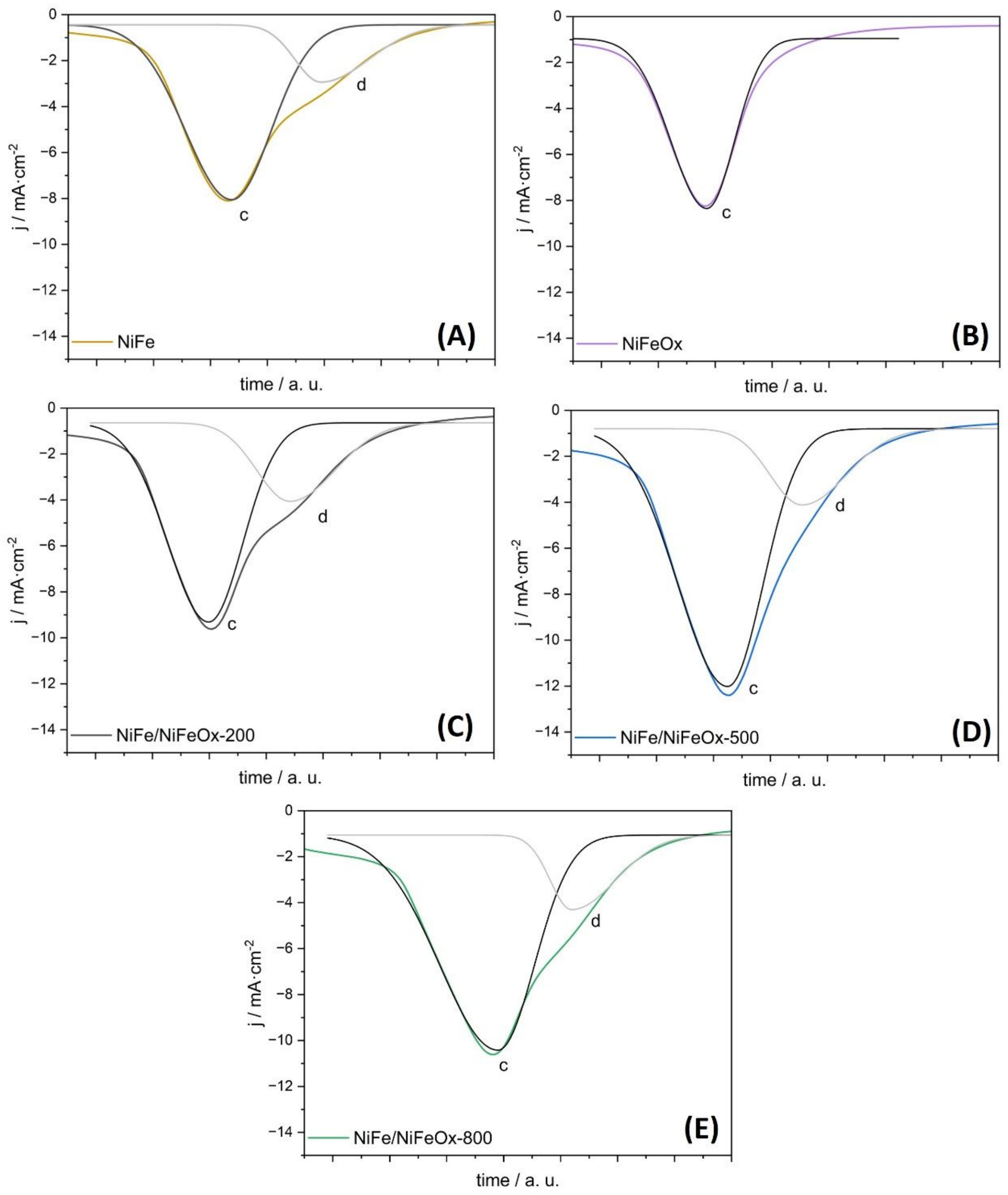

***Figure S13**. I vs t plots of cathodic signal from 200th cycle subtracted from CV conditioning process. Results have been integrated to determine the total charge employed during the reduction process. Deconvoluted "c" and "d" signals correspond to the processes summarized in **figure 1C**.*

The cathodic signal reported in **Figure S13** was deconvoluted in two signals (c and d) associated to the processes reported in the scheme of **Figure 1C** from the main manuscript. The current of both signals was integrated by time to obtain the total transferred charge (Q), which was used to determine the number of exchanged electrons using Faraday Law:

$$Q = n \cdot F \qquad \text{eq. S2}$$

Where *n* is the mols of electrons exchanged and F the Faraday's constant. As the number of electrons exchanged to reduce Ni^{3+} to Ni^{2+} is one, we can assume that the mols of transferred electrons is equivalent to the mols of Ni oxidized/reduced during the CV treatment.

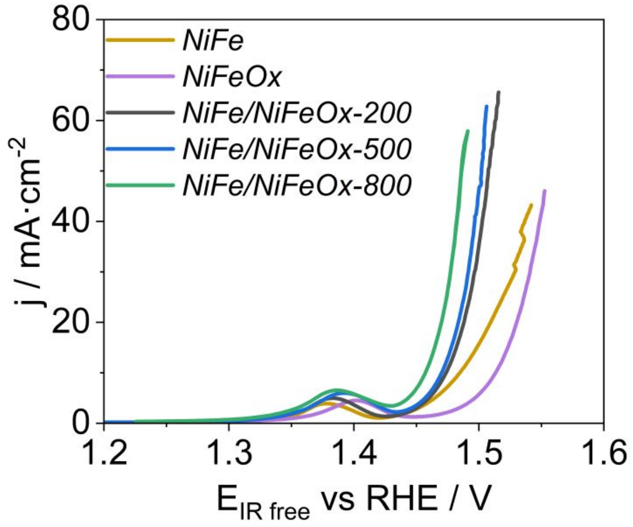

Figure S14. *LSV recorded after the ageing test performed in **figure 5E** of the main text.*

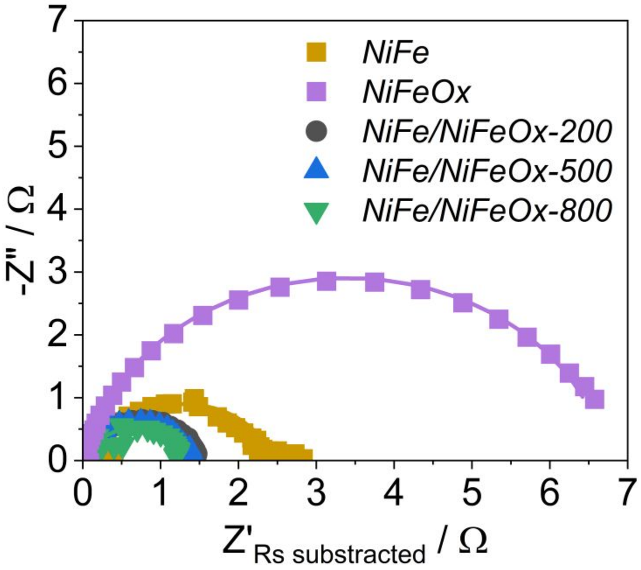

Figure S15. *Nyquist plots acquired at 1.53 V vs RHE after the ageing test performed as reported in **figure 5E** of the main text.*

These Nyquist plots are recorded under the same conditions than those reported in **Figure 5C** of the main text (i.e, 1.53 V).

Supporting information SI9.- Electrochemical characterization of samples in single cell devices.

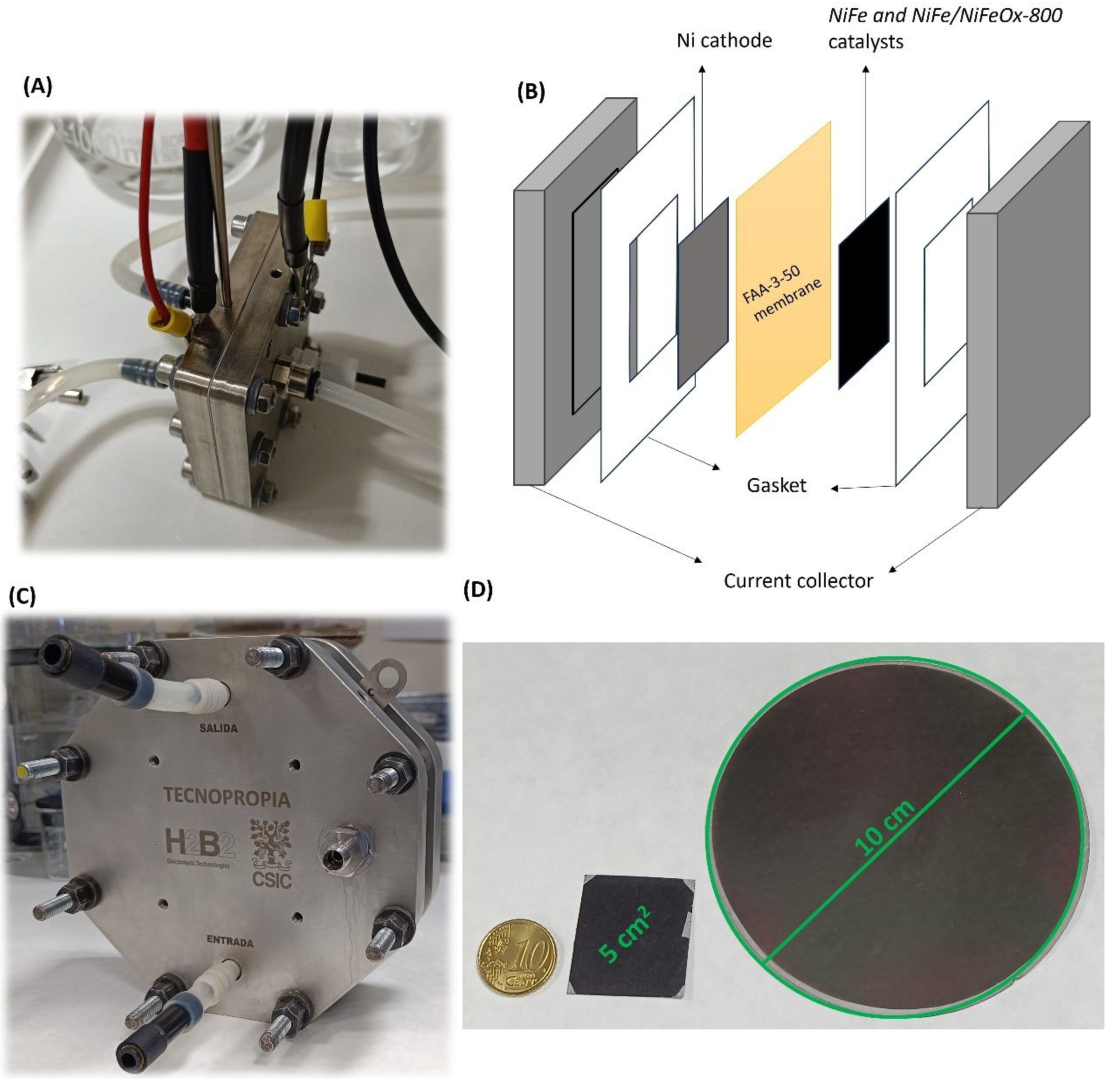

Figure S16. *(A) Picture of the 5 cm^2 electrode area single AEMWE cell employed to characterize the electrochemical performance of bilayer catalyst coated PTL anodes considered in this work. (B) Assembling scheme. (C) Picture of the scaled 64 cm^2 active electrode area employed for large-scale studies. (D) Illustration of the two electrode sizes employed in the different tests.*

Table S6. *Resistance values determined from the fitting analysis of Nyquist plots acquired in full cell configuration and reported in **Figure 7C** of the main text. They correspond to PTL/NiFe and PTL/NiFe/NiFeOx-800 anodes. The third column shows the R_{Cell} increment after the stability test at 0.6 A·cm^{-2} reported in **Figure 7B**.*

**Single Cell**	**R_{Cell} (Ohm)**		
**anode**	**initial**	**after aging**	**ΔR_{Cell} (%)**
PTL/NiFe	0.17	0.34	100
PTL/NiFe/NiFeOx-800	0.19	0.30	58
